# Efficacy of interactive video gaming in older adults with memory complaints: A cluster-randomized exercise intervention

**DOI:** 10.1371/journal.pone.0252016

**Published:** 2021-05-25

**Authors:** Udhir Ramnath, Laurie Rauch, Estelle Victoria Lambert, Tracy Kolbe-Alexander

**Affiliations:** 1 Division of Exercise Science and Sports Medicine, Department of Human Biology, Faculty of Health Sciences, University of Cape Town, Cape Town, South Africa; 2 School of Health and Well-Being, University of Southern Queensland, Ipswich, Australia; 3 School of Human Movement and Nutrition Sciences, The University of Queensland, Brisbane, Australia; Iwate Medical University, JAPAN

## Abstract

**Purpose:**

The effects of aging on physical and mental health may be ameliorated by regular participation in physical activity (PA). There is also evidence for the benefits of various training modalities on cognition and functional ability in older adults. The aim of this study was to compare effects of a 12-week active video gaming intervention (X Box Kinect Sports) to conventional multimodal supervised exercise on fitness, functional ability and cognitive performance in older adults with memory complaints.

**Methods:**

Participants (n = 45, 72±5 yrs.) were recruited from 6 retirement homes and cluster-randomized into the Interactive Video Gaming (IVG) group (N = 23) or Conventional Multimodal (CM) group (N = 22), meeting 2 x 1 hour sessions, weekly for 12 weeks. Pre-post measures included: 6 min walk, timed up and go, dynamic balance, functional reach, Mini-Mental State Examination, N-back Task and the Modified Stroop task.

**Results:**

The IVG group demonstrated significant improvement in the total number correct responses on the Stroop task (P = 0.028) and for average reaction time of correct colour-words (P = 0.024), compared to the CM group. Functional ability improved significantly in the IVG group, including the 6-min walk (P = 0.017), dynamic balance (P = 0.03), timed up and go (P<0.001) and functional reach (P<0.0010).

**Conclusion:**

An active interactive video gaming intervention was more effective than conventional multimodal exercise in improving executive and global cognitive performance and functional capacity in older adults with subjective memory complaints.

**Trial registration:**

Pan African Clinical Trial Registry—PACTR202008547335106.

## Introduction

The decrease in cognitive performance that occurs with aging leads to decreased health status and quality of life, and may lead to older adults experiencing memory complaints, associated with poor executive function and delayed recall [1]. Memory complaints, which may later lead to mild cognitive impairment or dementia have been shown to be mitigated or reduced by multifactorial interventions which include a change in diet, stress reduction techniques such as meditation and participation in regular exercise [2].

Previous research has shown minor improvements, as a function of participation in physical activity and exercise, in cognitive and brain health in older adults [3, 4]. The aging process leads to grey and white matter atrophy, especially in the prefrontal cortex and hippocampus. Colcombe and colleagues have identified that improved cardiorespiratory fitness is associated with a reduced loss of grey and white matter in temporal, frontal and prefrontal regions of the brain [5]. In other studies, physical activity has been shown to improve cerebral oxygen uptake as well as increase Brain-Derived Neutrophic Factor (BDNF). This in turn, enhances cerebral plasticity, cell proliferation as well as synaptogenesis in the hippocampus which plays an important role in memory formation [6]. Furthermore, physical activity also increases dopaminergic activity in the basal ganglia which also promotes enhancements in memory. Research on conventional exercises has also shown benefits for physical function in older adults [7], however, to our knowledge, minimal research studies have been conducted identifying the effects of conventional multimodal supervised exercises on cognitive performance in older adults with memory complaints.

Further, recent studies have identified that a combination of physical and mental exercises can improve cognitive function in healthy older adults. Shah et al. 2014 showed that the combination of home-based physical activity and computerized cognitive stimulation over 16 weeks improved verbal episodic memory in healthy older adults [8]. Similarly, Nishiguchi and colleagues showed significantly greater improvements in executive function and memory in community-dwelling older adults after 12 weeks of a combined physical and cognitive intervention compared to the usual care control group [9].

Emerging research suggests that interactive video gaming may enhance physical characteristics such as postural balance and muscle strength that may transfer to other cognitive domains [10, 11]. Interactive video gaming requires the use of body movements to control the onscreen character’s movement, therefore allowing the individual to increase their energy expenditure while participating in games that were traditionally sedentary in nature [12]. Interactive video gaming is known to develop and improve spatial awareness, attention span (amount of time an individual can concentrate on a task without becoming distracted), plan actions and understand spatial constraints [13]. It also has the ability to teach the player to respond to visual feedback and create a cognitive map of their bodily movement in relation to the game [14]. Active video gaming may also directly improve cognitive functioning, especially those related to executive control skills, by providing aerobic activity when playing. These improvements can be explained by physiological changes that occur with increased levels of physical fitness which improves cognitive performance due to increased cerebral circulation and neurotransmitter availability [15]. Further to these improvements, active video gaming also involves a cognitive component that may enhance cognitive function. A study conducted by Shatil in 2013 found that the use of aerobic exercise combined with cognitive training may have additive effects that could prevent cognitive decline and stimulate neurogenesis through independent pathways that may be complimentary to each other [16]. Due to the player being a short distance from the screen, it also requires the player to incorporate cognitive skills in the form of visual-spatial skills, hand-eye or foot-eye coordination and quick reactions to play the specific games successfully. Therefore, IVG is different from traditional exercise, as it not only requires physical skill and ability, but also has a complex cognitive component in order to successfully play the game [17].

This is a growing area of research investigating the effects of an interactive video gaming intervention on cognitive performance and functional ability in individuals with various cognitive conditions such as mild cognitive impairment and dementia [18, 19]. In 2019, Palac and colleagues conducted a study to evaluate the feasibility and efficiency of an IPAD-enhanced aerobic virtual reality intervention over 10 weeks in 27 individuals aged 45 to 62 years [20]. Individuals were either randomized into an IPAD-enhanced aerobic exercise group (n = 14) or an aerobic exercise control group (n = 13) and participated twice a week in their respective programs. Post intervention, the iPad experimental group significantly improved on an attentional-control cognitive task compared to the control group (p<0.05) [20]. Although Palac et al. 2019 found improvements on a cognitive task between groups post intervention, the iPad intervention used in their study may differ to other interactive video gaming consoles such as the Xbox Kinect or Nintendo Wii as well as their respective gaming software.

Previous studies have also illustrated benefits of interactive video gaming on cognitive performance measures such as executive control, attention, reaction time, processing speed and memory in healthy functioning older adults as well as older adults diagnosed with mild cognitive impairment [10, 18]. However, few studies have investigated whether active gaming can improve cognitive function in individuals with subjective memory complaints. There are no other studies to our knowledge, comparing the benefits of active video gaming to conventional multimodal supervised exercise in older adults with self-reported and objectively measured memory complaints.

Therefore, the aim of this research study was to determine the effects of a 12-week active gaming intervention, using the X-Box 360 gaming console with Kinect Sports games, on cognitive performance in South African older adults with subjective and objective memory complaints. This intervention was compared to a conventional, multimodal supervised exercise program which served as the comparison group. We hypothesized that the individuals in the intervention group would show greater improvements in measures of cognitive function and functional ability than the comparison group.

## Methods

### Study design

Participants were recruited from six different retirement homes in Cape Town, South Africa for this quasi-experimental, cluster-randomized exercise intervention. Homes were randomly allocated to either the intervention group (Interactive video gaming) or to the comparison group (conventional multimodal supervised exercise program) for 12 weeks. All retirement homes were selected from similarly matched social and economic communities. All groups met for one hour, two days per week, for 12 weeks. Measurements were taken after randomization (baseline) and at the end of the 12 week program. All measurements, IVG sessions (intervention group) and CM supervised exercise sessions (comparison group) were conducted by the same researcher. The researchers were not blinded during the study. After the details of the study were explained, participants signed a written informed consent. The present study was approved by the University of Cape Town, Human Research Ethics Committee (HREC REF: 529/2015). In addition, the study has been registered with the Pan African Clinical Trial Registry (Trial identification number: PACTR202008547335106).

### Study procedures

An information session was conducted for each participant prior to baseline testing to explain the procedure of the testing routine as well as to describe the nature of the physical activity that was administered over the 3 month period. During the first session, potential participants went through a cognitive screening criterion and answered a physical activity readiness questionnaire (PAR-Q) to determine if they were eligible to participate in the research study. Eligible participants for the research study were then invited to the Sports Science Institute of South Africa for baseline measurements. The baseline visit included: 1) Completion of the demographic, health and wellness questionnaire; 2) Clinical measurements including height, weight, body mass index, waist circumference and body fat percentage; 3) Cognitive performance tests which included the Mini-Mental State Examination (MMSE), the Modified Stroop task and the “N-Back” test and; 4) Physical Function test which included the 6-minute walk test, dynamic balance, timed up and go and functional reach. All clinical measurements, cognitive performance and physical function tests were completed at baseline and post intervention. The first intervention session was conducted within a week of baseline testing. An attendance register was used to record each session to determine adherence. The intensity of the IVG and CM supervised exercise sessions were self-monitored by the use of the Borg’s RPE scale (1–10). Individuals were asked to provide feedback during their respective program sessions on their rate of perceived exertion on a scale from 1 to 10 and the researchers ensured that the individuals participated within their limit.

### Participants selection criteria

#### Recruitment

Individuals were recruited from the Cape Peninsula Organization for the Aged (CPOA) retirement homes. The housing manager of the Cape Peninsula Organization for the Aged was contacted via email to set up a meeting to discuss the nature of the research study. Once permission was granted for the study, information posters about the study were pinned on notice boards around the respective retirement homes. A total of 51 residents showed interested in the research study and were screened for eligibility. All individuals resided in retirement homes of similar socio-demographic and economic status. The individuals in each program were blinded to the researchers’ hypothesis.

#### Inclusion and exclusion criteria

Individuals aged 65 years and older with subjective and objective memory complaints and residing in a retirement home were eligible. We adapted the Petersen criterion for MCI to determine subjective and objective memory complaints in older adults not clinically diagnosed with MCI [21]. Individuals were eligible to participate in the study if they met all 5 of the criteria (Subjective memory complaints [22], Objective memory impairment [23], Intact daily function [24], Normal cognitive function (Adapted Telephone Interview for Cognitive Status (TICS)) and Non-demented [25]) described in Table 1:

**Table 1:** Cognitive function screening criteria.

*Criteria*	*Assessment Questions*	*Scoring*
**1) Subjective Memory Complaint [22]**	1.1 Do you have a memory complaint?	1) Yes
		2) No
	1.2 Strawbridge scale of cognition assessment questions:	Participants selected one possible answer from the list below for each question:
	a) Do you have difficulty paying attention	1) Rarely or never had the problem in the last 12 months
	b) Do you have trouble finding the right word	2) Sometimes had the problem
	c) Do you have difficulty remembering things	3) Often had the problem
	d) Do you forget where things are put	4) Very often had the problem
		Participants were classified as having a self-reported memory complaint if reported that they have memory complaints (**Yes**) in addition to answering ‘**Sometimes had the problem**’ in at least of two questions on the Strawbridge scale of cognition.
**2) Objective Memory Impairment [23]**	2.1 10 Word Learning Test	Scoring based on the number of words recalled per a trial with a minimum score of 0 and a maximum score of 10
	a) Trial 1	Objective Memory Impairment = Scored less than or equal to 5 on the 10 word learning test delayed recall
	b) Trial 2	
	c) Trial 3	
	d) Trial 4 Delayed Recall	
	(5 minutes later)	
**3) Intact Daily Function [24]**	3.1 Groningen Activity Restriction Scale was used to assess activities of daily living (ADL) (11 questions were administered)	Participants selected one possible answer from the list below for each question:
		**POINTS**
		Yes I can do it fully independently without any difficulty (1 point)
		Yes I can do it fully independently but with some difficulty (2 points)
		Yes I can do it fully independently but with great difficulty (3 points)
		No I cannot do it fully independently. I can only do it with someone’s help (4 points)
		No I cannot do it at all. I need complete help (4 points)
		Intact ADL function if they reported no disabilities on the ADL items except for “Care of feet and toe nails”
**4) Normal Cognitive Functioning [25]**	4.1 Telephone Interview for Cognitive Status (TICS) was used to assess mental status, orientation, concentration, calculation, memory, judgment and reasoning. We adapted the TICS so that it could be administered face-to-face.	Score Range: 0–41
		Score greater than or equal to 19 on the TICS was categorized as normal cognitive status
**5) Non-Demented**		Individuals regarded non-demented with a score of greater than or equal to 19 on the TICS

Older adults who had been previously diagnosed with a stroke, Parkinson’s disease, balance disorders, impaired vision or had uncontrolled conditions such as hypertension (blood pressure more than 160/90 and not on medication) or diabetes (not on medication) were excluded from the study. Those with orthopaedic injuries which inhibited normal locomotion were also excluded.

The Physical Activity Readiness Questionnaire (PAR-Q) was used as the first line of screening for participant’s entry into the study [26]. Those who were identified as being at an increased risk for coronary artery disease, according to the American College of Sports Medicine (ACSM) criteria, required consent from a doctor to allow them to participate [26]. Participants were classified as increased risk if they had more than two cardiovascular risk factors and were symptomatic for or diagnosed with cardiovascular, pulmonary and / or metabolic disease. The cardiovascular disease risk factors include: family history of death by cardiovascular disease (sudden death by father or first degree relative before 55 years of age and mother or other first degree female before 65 years of age); current smoker or those who quit within the previous 6 months and not participating in at least 30 minutes of moderate intensity physical activity for at least 3 days a week for at least 3 months. In addition, men with a waist circumference of > 102cm and women greater than 88cm; hypertension (blood pressure >140mmHg / 90mmHg on two separate occasions); total cholesterol > 5.18mmol and fasting plasma glucose > 5.50mmol [26].

### Interactive video games (IVG) group

Participants in the intervention group attended two, one hour interactive video game (IVG) sessions per week. The IVG sessions took place at the respective retirement homes for the duration of the 3 month program. The X-Box Kinect Sports video gaming software was used and comprises of 6 games namely: ten-pin bowling, boxing, track and field, table tennis, beach volleyball and soccer. Each game is described in the Table 2.

**Table 2:** Description of interactive video games.

Name	Fitness Component	Brief Description
Ten-pin bowling	Shoulder flexibility and balance	Individuals were required to reach either to the left or right hand side to pick up a ball before swinging their arm forward to bowl. This game will require good mobility and balance. The aim of this game was to knock over as many pins as possible
Boxing	Bilateral arm flexibility, agility, endurance and balance	The game required individuals to move in all directions, use both hands to punch forward and block, both at head and body height. The aim was to either score more points than the opponent during the fight or to knock the opponent out.
Track and Field	All-round flexibility, endurance, agility lower body strength and balance	The track and field is a collection of 5 separate events namely: sprint, javelin, long jump, discus and hurdles. Individuals jogged on the spot to run / sprint, jump up to clear hurdles or jump forward for long jump as well as performed the relevant arm movements to throw a javelin or discus. The aim was to score as many points in each of the respective events.
Table Tennis	All-round flexibility and agility	Individuals were required to reach either to the left or right hand side to pick up a paddle. They then mimicked the movements of table tennis to hit the ball. The aim was to score 11 points first.
Soccer	Lower body strength and agility	In this game, individuals played as attackers and defenders. As an attacker, the individual was required to kick the ball in order to pass or shoot. As a defender, the individual needed to move side to side to block passes and use their body to block shots. The aim was to score more goals than the opponent.
Beach Volleyball	Upper body strength and flexibility	This game required the use of both hands. To serve, the participant made an upward throwing motion with one hand and then a swinging motion with the other hand. Passing, returning or spiking the ball was done by mimicking volleyball movements. The aim was to score 7 points first.

The participants played each of the 6 games at each session with a partner and two pairs played (n = 4) per session. The first pair played the game for 15 minutes while the second pair rested after which the roles were reversed. Therefore, each pair played for a total of 30 minutes and had 30 minutes break per session.

Each session was supervised by an exercise physiologist who acted as a ‘spotter’ during the gaming session to minimize the risk of any injury or to help regain balance if needed. This was done by standing slightly behind and between the individuals during the gaming routine. An attendance register was used to record the number of sessions each participant attended over the 12 weeks. No adverse incidents and no falls occurred during the 12 weeks.

### Conventional multimodal (CM) group

The comparison group met for one hour, twice per week, for 12 weeks and participated in group-based low intensity conventional multimodal supervised exercise sessions.

The exercise program consisted of a combination of standing and seated exercises. The exercise sessions comprised of a 10 minute warm up, 30 minutes of strength training, followed by 10 minutes of proprioceptive exercises and 10 minutes of cooling down. All exercises were performed using body weight only for the first 6 weeks. The intensity of the upper body strength training exercises for weeks 7 to 12 were increased by using plastic soft drink bottles (500ml) filled with sand as weights. Participants enjoyed a 45 second break after each set of exercises for the duration of the 12 weeks. Each set consisted of 15 repetitions per an exercise. The sessions were supervised and coordinated by an exercise physiologist. No adverse incidents and no falls occurred during the 12 weeks. Table 3 describes the exercise program in more detail.

**Table 3:** Exercise program.

Program Component	Duration	Type of Exercises
1) Warm Up	10 minutes	1) Chair marching while seated
		2) Lateral circular arm movements while standing
2) Strength Training	30 minutes (Two sets of 12 repetitions were performed for each strength exercise)	Lower body exercises:
		1) Seated straight leg raise
		2) Standing heel raises
		3) Standing knee raises
		4) Seated ankle circles
		5) Seated alphabet tracings
		Upper body exercises:
		1) Seated bicep curls
		2) Frontal arm raises (Standing)
		3) Lateral arm raises (Standing)
		4) Single arm shoulder press (Standing)
		5) Seated horizontal chest press
3) Proprioceptive Exercises	10 minutes	1) Double leg balance with eyes open and then with eyes closed
		2) Supported single leg balance with eyes open and then closed
4) Cool down	10 minutes	1) On the spot slow walking
		2) Stretching

### Measurements

#### The demographic, health and wellness questionnaire

An interviewer-administered questionnaire was conducted to obtain information on demographics and health (section 1) and weekly physical activity (section 2). The first section was used to obtain information about the participant’s age, gender, marital status, past and present occupation, perceived health status and highest level of educational achievement. Participants were also asked to report any previous disease diagnosed by a medical doctor including the treatment and medication received.

The second section of the questionnaire was based on Part One of the Yale Physical Activity Survey for older adults [27] and was used to assess physical activity in five domains, namely, household, yard work, care giving, recreation and exercise. Participants were required to recall the amount of time spent in each of these five domains for various activities. Each activity was assigned an intensity (kcal) as described by DiPietro [27], and subsequently multiplied by the time spent to calculate kcal/week. Individual weekly energy expenditure was calculated for each of the activity domains and then summed to estimate total weekly energy expenditure. This questionnaire was only administered at baseline to determine habitual physical activity levels amongst participants both groups.

#### Clinical measurements

Height was measured to the nearest centimetre using a wall mounted measuring tape and with participants unshod (Detecto). Participants’ body mass was measured using a calibrated electric scale and recorded to the nearest 0.5kg. Body Mass Index (BMI) was calculated as body mass (kg) divided by height (m) squared (kg/m^2^). Waist circumference was recorded at the level of the umbilicus [26].

A Harpenden calliper was used to measure skin fold thickness at four anatomical sites: sub-scapular, supra-iliac, triceps and biceps. The sum of the four skin fold sites was then be substituted into the Durnin and Womersley skin fold equation which was used to calculate percentage body fat [28]. All clinical measurements were conducted at baseline and post intervention.

#### Cognitive performance tests

*The Mini-Mental State Examination (MMSE)*. The MMSE is an interview administered cognitive outcome measure of global cognitive function and was used to assess cognitive impairment [29]. This test consists of 11 questions concerning orientation, registration, attention and calculation, recall and language. The test takes between 5–10 minutes to administer and the score obtained can range from 0 to 30 [29].

*The Modified Stroop task*. The modified version of the original Stroop task was adapted to enable participants to use a computer keyboard instead of responding verbally [30]. Words, 2cm high, appeared in the centre of a computer screen on a black background. A total of 32 words (24 colour (incongruent) and 8 grey (neutral) words) appeared randomly in one of five colours (red, blue, green, yellow or grey) every 3 seconds (displayed for 400ms, followed by a black screen for 2600ms, allowing the participant time to respond) on the computer screen. The words were either presented in grey ink (neutral) or in a colour ink incongruent with the meaning of the word, i.e. blue in red colour, or yellow in green colour, but never green in green colour or red in red colour (congruent), etc. The participants were asked to respond as quickly and as accurately as possible by pressing one of four response buttons to indicate either the colour of the word (if the word is written in colour ink), or the word itself (if the word is written in grey ink). The word cues consisted of 4 colour-words (red, blue, green and yellow), appeared.

Participants were allowed two, 2-minute familiarization trials, before completing a third trial. Data for all 3 Modified Stroop tasks were recorded, but only the third trial was used in the data analysis. During the post intervention testing period, each participant was allowed one familiarization trial and one final trial. Both attempts were recorded, however, only the final trial was used in the data analysis.

*The “N-Back” task*. The *N*-Back task (“n” represents the level number; example: 1-back, 2-back) has previously been used to assess working memory (WM) [31]. During the *n*-back task, participants were presented with a sequence of letters, one at a time, on an Apple I-pad screen and requested to compare the current letter to the one presented “*n*-back” in the sequence. Participants used the index finger of their dominant hand to respond by pressing the middle of the Ipad screen when the current letter matched the letter that was *n-back* in the sequence. The letter sequence was presented randomly in a fixed location in the centre of the computer screen for duration of 600m.s with a 2600m.s inter-stimulus interval.

Individuals started with the 0-back task. In the 0-back condition, individuals were required to respond when an “X” appeared on the screen only. When performing the 2-back task, the current letter is a target when it matches the letter presented two positions ago. For example, in the sequence “R-I-T-A-?” the individual should respond by pressing the middle of the screen if the 5^th^ letter in the sequence is a “T” because it would match the letter occurring two places prior. During this study, only the 0-back, 1-back and 2-back task were used. During the 0-back trial, a total of 135 targets were presented. In the 1-back trial, a random number of targets between 60 and 70 were presented, and in the 2-back trial, a random number of targets between 50 and 60 were presented. Each N-back level score was displayed as a percentage of the total number correct targets achieved. Individuals were allowed one familiarization trial (0-back task) and one final trial for each *n*-back task (1-back and 2-back). All trials were be recorded and used for data analysis.

#### Physical function tests

*6-minute walk test*. Individuals were asked to walk as far as possible in six minutes [32]. A 70m indoor track was used, with one chair positioned at the start and one at 35m, to give individuals an opportunity to sit and rest if needed. The total distance covered was recorded in metres.

*Dynamic balance*. The individuals were asked to walk 10 metres along a strip of tape using the tandem gait. The tandem gait involves walking heel to toe, i.e. when the right foot is in front, the left heel will move forward touching the right toe and vice versa in a straight line along the strip of tape [33]. The time taken to complete the first 6 steps and to complete the 10 metres was recorded.

*Timed up and go test*. Individuals started in a seated position on a standard chair with no arm rests. On the command of “Go” they arose from the chair while keeping their arms across their chest, walked 3 metres, turned around and walked back to the chair and sat down. The time taken to complete the task was recorded [34].

*Functional reach*. Individuals were asked to stand next to a wall and reach as far as possible along a wall mounted yardstick without moving / taking a step [35]. Each Individual had two familiarization trials before completing three more trials. The mean value of the last three trials was recorded.

### Statistical analysis

STATISTICA 10 software package for Windows 10 was used for all the analyses (Stasoft, Inc., Tulsa OK, USA). Descriptive statistics were performed for the IVG and CM groups separately and data were represented as means and standard deviations. Frequency tables were used to determine demographic, health and wellness variables percentages. T-tests were conducted to identify significant differences between groups for baseline screening, ADL and demographic and health characteristics.

Analyses of variance for repeated measures (ANOVA) were used to detect differences between groups for changes in continuous variables (cognitive performance and physical function) at baseline and post intervention. ANCOVA was conducted before and after co-varying for age and physical activity. All models were adjusted for site level clustering appropriate for an experimental cluster-randomized intervention study. The level of significance for all statistical analyses was set at p < 0.05. Partial eta^2^, defined as the ratio of variance accounted for by an effect and that effect plus its related error variance within an ANOVA study [36], was displayed as measures of effect size for each cognitive and physical function outcome variable. The sample size was estimated using G*Power software (Version 3.1.9.2) [37, 38]. The sample size was determined using a priori power calculation (repeated measures, within-between interaction, medium effect size = 0.6, power of 0.80, 2-sided α-error, 2 groups, 2 measurements) based on the primary outcome of the Mini-Mental State Examination from a comparable study [39]. The required sample size calculated with an additional 20% increase per a group taking into account drop outs was 12 participants per a group.

## Results

**Fig 1:**
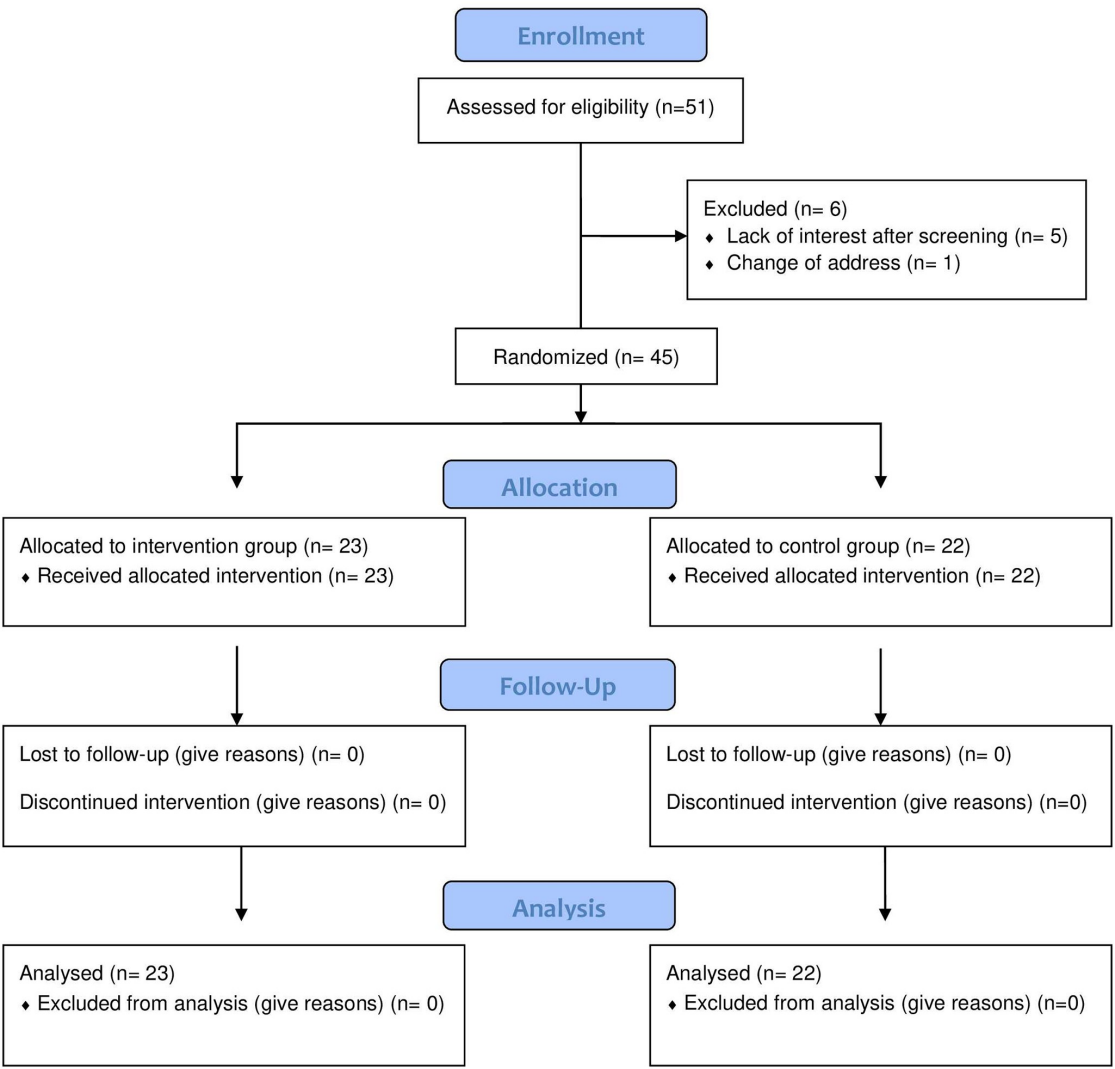
Enrollment and allocation flowchart.

A total of 51 people volunteered to participate in the study and were initially screened (Fig 1). All individuals met the inclusion criteria to participate in the study, however, 6 participants chose not to participate in the study due to a lack of interest after screening (n = 5) and change of address (n = 1). They did not complete any IVG or conventional multimodal sessions. The final sample size for the study was then reduced to 45 participants. All 45 participants presented for all tests and sessions throughout the study (Fig 1).

Participant baseline screening cognitive and ADL characteristics are presented in Table 4. All participants in both groups answered ‘Yes’ to having a memory complaint. The only difference between the two groups was the Groningen Activity Restriction Scale score, which was significantly better in the comparison group, suggesting that the comparison group had better function for activities of daily living. All the participants scored more than 19 on the adapted version of the TICS and were therefore considered to be non-demented.

**Table 4:** Baseline screening cognitive ability and ADL characteristics.

	Total (n = 51)	Intervention Group (n = 27)	Comparison Group (n = 24)	p-value
	Mean (±S.D)	Mean (±S.D)	Mean (±S.D)	
**Strawbridge Scale of Cognition (Score)**	1.8 (±0.25)	1.9 (±0.27)	1.8 (±0.23)	0.221
**10 W.L.T (Number)**:				
1) Immediate Recall 1	4.6 (±1.37)	4.3 (±1.40)	5.0 (±1.27)	0.069
2) Immediate Recall 2	5.4 (±1.25)	5.3 (±1.23)	5.5 (±1.28)	0.574
3) Immediate Recall 3	5.9 (±1.13)	5.9 (±1.11)	5.8 (±1.17)	0.773
4) Average Immediate Recall of 3 attempts	5.3 (±0.97)	5.2 (±1.04)	5.4 (±0.89)	0.331
5) Delayed Recall	2.8 (±1.70)	3.00 (±1.71)	2.5 (±1.69)	0.342
**GARS (Score)**	11.6 (±1.09)	12.0 (±1.34)	11.2 (±0.38)	0.004*
**TICS (Score)**	29.2 (±3.30)	29.4 (±3.33)	29.0 (±3.32)	0.693

10W.L.T– 10 Word Learning Test; GARS—Groningen Activity Restriction Scale; TICS—Telephone Interview for Cognitive Status;

S.D = Standard Deviation

Values are shown as mean ± standard deviation

*p<0.05

Participant’s demographic and health characteristics are presented in Table 5. The mean age of the participants was 72.4 ± 5.4 years with the comparison group being significantly older; therefore analysis was performed co-varying for age. The highest percentages of both groups are represented by individuals who are widowed. Majority of participants in the intervention (65.2%) and comparison (77.3%) group have achieved primary school education as their highest level of formal education. Hypertension was the most frequently reported medical condition in the intervention (78.3%) and comparison (68.2%) groups. Almost all of the participants were taking prescribed medication.

**Table 5:** Baseline demographic and health characteristics.

	Total (n = 45)	Intervention Group (n = 23)	Comparison Group (n = 22)
	Mean (±S.D)	Mean (±S.D)	Mean (±S.D)
**Age (Years)**	72.4 (±5.37)*	70.8 (±4.52)	74.14 (±5.8)
**Body Mass Index (kg·m**^**2**^**)**	29.1 (±5.66)	29.0 (±5.47)	29.16 (±6.0)
**Waist Circumference (cm)**	88.8 (±11.81)	89.3 (±11.51)	88.15 (±12.4)
**Percentage Body Fat (%)**	34.4 (±3.01)	34.4 (±3.40)	34.43 (±2.6)
**Total Weekly Physical Activity (Minutes)**	1143.6 (±975.31)	1149.0 (±1099.67)	1138.0 (±852.14)
**Total Weekly Energy Expenditure per (Kcal / Week)**	3467.8 (±2547.89)	3382.2 (±2392.90)	3557.2 (±2754.40)
**Marital Status**:			
1. Married	11 (24.4%)	4 (17.4%)	7 (31.8%)
2. Single	3 (6.7%)	2 (8.7%)	1 (4.6%)
3. Divorced	10 (22.2%)	4 (17.4%)	6 (27.3%)
4. Widowed	21 (46.7%)	13 (56.5%)	8 (36.4%)
**Highest Level of Education**:			
1. Primary School	32 (71.1%)	15 (65.2%)	17 (77.3%)
2. Secondary School	2 (4.4%)	2 (8.7%)	-
3. Tertiary	11 (24.4%)	6 (26.1%)	5 (22.7%)
**Health Status**:			
1. Hypertension	33 (73.3%)	18 (78.3%)	15 (68.2%)
2. High Cholesterol	24 (53.3%)	13 (56.5%)	11 (50.0%)
3. Diabetes Mellitus	14 (31.1%)	10 (43.5%)	4 (18.2%)
4. Asthma	3 (6.7%)	3 (13.0%)	-
5.PeripheralVascular Disease	1 (2.2%)	1 (4.4%)	-
6. None	3 (6.7%)	2 (8.7%)	1 (4.6%)

S.D = Standard Deviation

Values are shown as mean ± standard deviation

Marital status, Education and Health status are represented as a number (percentage)

*p<0.05

Displayed in Table 6 are the cognitive performance changes after 12 weeks between the intervention and comparison group. The intervention group significantly improved in the total number of correct responses on the Stroop task when compared to the comparison group (p = 0.028). Significant interaction effects between groups were found after 12 weeks for the Stroop task correct colour word percentage (p = 0.011) and average reaction time of correct colour words (p = 0.024), with the intervention group showing significantly greater improvements. The intervention group also significantly improved in the Mini-Mental State Examination compared to the comparison group (p = 0.005). The ANOVA showed no significant group or time main effects or interaction effects for any of the other cognitive performance variables. Co-varying for age and physical activity did not change the interpretation of results, nor differences between groups. Interference scores were calculated for both groups by subtracting the average reaction time of incongruent words from the average reaction time of neutral words. The intervention group’s interference score (average reaction time of correct incongruent—average reaction time of correct neutral) was 20.4 at baseline and 237 at 12 weeks. Similarly, the comparison group’s interference score was 131.8 at baseline and 111.8 at 12 weeks.

**Table 6:** Changes in cognitive performance in the intervention and comparison groups.

Variable & Assessment Period		Intervention Group (n = 23)	Comparison Group (n = 22)	Group Effect *(p-value)*	Time Effect *(p-value)*	Group by Time Effect *(p-value)*	Partial eta^2^
***STROOP TASK***:		**Mean (± S.D)**	**Mean (± S.D)**				
Total Correct Responses (%)	Baseline	64.2 (±19.90)	70.8 (±19.93)	0.956	0.970	0.028*	0.1095
	12 Weeks	80.7 (±20.96)	74.8 (±20.97)				
Average reaction time of all Correct Responses (m.s)	Baseline	1717.5 (±226.46)	1635.5 (±226.73)	0.886	0.736	0.092	0.0660
	12 Weeks	1594.7 (±237.97)	1660.3 (±238.23)				
Correct Grey (Neutral) Words (%)	Baseline	60.0 (±32.23)	61.7 (±32.27)	0.631	0.432	0.725	0.0029
	12 Weeks	74.3 (±25.42)	79.7 (±25.47)				
Average reaction time of Correct Grey (Neutral) Words (m.s)	Baseline	1713.2 (±661.78)	1702.3 (±662.57)	0.787	0.877	0.901	0.0003
	12 Weeks	1770.3 (±419.01)	1725.6 (±419.46)				
Correct Colour (Incongruent) Words (%)	Baseline	65.7 (±22.06)	73.8 (±22.09)	0.897	0.718	0.011*	0.1436
	12 Weeks	82.9 (±21.92)	73.2 (±21.95)				
Average reaction timeof Correct Colour (Incongruent) Words (m.s)	Baseline	1692.8 (±240.70)	1570.5 (±240.99)	0.744	0.516	0.024*	0.1161
	12 Weeks	1533.3 (±261.28)	1613.8 (±261.58)				
Average reaction time of total number of Mistakes (m.s)	Baseline	1559.2 (±561.93)	1432.2 (±562.57)	0.851	0.411	0.395	0.0172
	12 Weeks	1434.9 (±685.56)	1503.6 (±686.35)				
Average reaction time of Incorrect Colour (Incongruent) Words (m.s)	Baseline	1493.8 (±584.28)	1423.3 (±584.94)	0.415	0.366	0.116	0.0579
	12 Weeks	1155.7 (±731.41)	1487.9 (±732.27)				
Average reaction time of Incorrect Grey (Neutral) Words (m.s)	Baseline	1007.5 (±898.69)	705.3 (±899.72)	0.133	0.261	0.972	0.0000
	12 Weeks	994.3 (±896.53)	705.8 (±897.56)				
***N-BACK TASK***:							
0-Back Score (%)	Baseline	97.1 (±2.59)	96.6 (±2.58)	0.164	0.901	0.409	0.0163
	12 Weeks	98.6 (±1.77)	97.5 (±1.74)				
1-Back Score (%)	Baseline	93.7 (±6.76)	93.5 (±6.75)	0.640	0.436	0.527	0.0096
	12 Weeks	96.0 (±5.66)	94.6 (±5.68)				
2-Back Score (%)	Baseline	59.8 (±27.24)	51.9 (±27.30)	0.511	0.896	0.286	0.0270
	12 Weeks	73.8 (±18.13)	73.7 (±18.15)				
***MINI MENTAL STATE EXAMINATION***:	Baseline	24.6 (±2.69)	25.0 (±2.67)	0.242	0.285	0.005*	0.1751
	12 Weeks	27.3 (±2.49)	25.3 (±2.49)				

*p<0.05;

ANOVA for repeated measures, co-varying for age and baseline physical activity (YPAS).

Displayed in Table 7 are the functional ability changes after 12 weeks between the intervention and comparison group. There was an interaction effect for the 6 minute walk test, with the intervention group improving significantly compared to the comparison group (p = 0.017). Dynamic balance also improved significantly in the intervention group in when compared to the comparison group (p = 0.030). In addition, significant improvements in the intervention group were also identified for the timed up and go test (p = 0.000) and the functional reach test (p = 0.000). The ANOVA showed no significant group effects for any variables, however, a main effect of time was identified for the 6 minute walk test, indicating that both groups improved significantly. An attendance register was taken at each session for both groups. All participants (n = 45) for both groups completed 24 one hour sessions over the 12 week intervention period.

**Table 7:** Changes in functional ability in the intervention and comparison groups.

Variable & Assessment Period		Intervention Group (n = 23)	Comparison Group (n = 22)	Group Effect *(p-value)*	Time Effect *(p-value)*	Group by Time Effect *(p-value)*	Partial eta^2^
		Mean (± S.D)	Mean (± S.D)				
**6 Minute Walk Test (m)**	Baseline	466.7 (±79.13)	491.8 (±79.27)	0.694	0.039*	0.017*	0.1283
	12Weeks	513.7 (±83.45)	507.7 (±83.49)				
**Dynamic Balance 10m Test (s)**	Baseline	46.4 (±13.91)	46.5 (±14.07)	0.246	0.916	0.030*	0.1070
	12 Weeks	35.7 (±11.03)	43.8 (±11.26)				
**Timed Up & Go Test (s)**	Baseline	7.4 (±1.40)	7.2 (±1.41)	0.212	0.945	0.000*	0.3507
	12 Weeks	6.2 (±1.24)	7.4 (±1.27)				
**Functional Reach Test (cm)**	Baseline	80.8 (±6.52)	83.3 (±6.52)	0.651	0.520	0.000*	0.3471
	12 Weeks	85.9 (±4.56)	82.0 (±4.55)				

*p<0.05;

ANOVA for repeated measures, co-varying for age and baseline physical activity (YPAS).

## Discussion

The aim of this research study was to determine the effects of a 12-week active gaming intervention, using the X-Box 360 gaming console with Kinect Sports games, on cognitive performance in South African older adults with subjective and objective memory complaints. We hypothesized that participants in the interactive video gaming group would show significantly greater improvements in measures of cognitive performance and functional ability compared to the comparison group. Our main finding supports this hypothesis as the data show that both the Stroop task and the Mini-Mental State Examination scores improved significantly in the IVG group, while there were no changes in the comparison group.

In our study, the video gaming intervention group showed significant improvements over time in the total correct number of responses on the Stroop task as well as the total correct colour word responses and average reaction time of correct colour words. Previous studies have shown that various forms of exercise training such as cardiovascular training, resistance training and balance training can alter brain plasticity which is associated with improvements in cognitive performance [40, 41]. The increase in availability of oxygen for cerebral activity could be accountable for the improvement in neurocognitive performance that arises with fitness training. Therefore in our study, we speculate that the improvement in executive function on the Stroop task with interactive video gaming may be due to the improvements in cardiovascular fitness with consistent participation. This may indicate that older adults who participate in interactive video gaming two or more times a week may be able to improve their cardiovascular fitness and thereby assist in improving their cognitive performance. Although there was a significant difference between both groups in the total correct responses on the Stroop task, the comparison group showed an improvement post intervention compared to baseline. In the past, studies have shown that exercise training can improve cognitive performance; however, not many studies have utilized interactive video gaming to show improvements in executive function over duration of 12 weeks in individuals with subjective memory impairment. Our findings are supported by Maillot and Perrot. 2012 who evaluated the effects of Interactive video-game training on cognitive and physical function in older adults [10]. Individuals participated in interactive video gaming for an hour twice a week using the Nintendo Wii gaming console and Wii sport games for 12 weeks while individuals in the control group remained sedentary. Cognitive performance was assessed using a wide variety of neuropsychological tests such as the Stroop task, Digit Symbol Substitution test and Trail-making test to measure executive function as well as other cognitive tests to assess attention, visuospatial function and processing speed. Participants in the intervention group improved significantly more than those in the sedentary control group in cognitive measures of executive function and processing speed [10].

Another study by Anderson-Hanley et al. 2012 also found similar results to our study by illustrated the effects of 3 months of either 3 dimensional virtual reality cycling tours versus traditional stationery cycling, on cognitive function in older adults [19]. Individuals (n = 79) were randomized and participated in either 3 dimensional virtual reality cycling tours (cyber-cycling) for 45 minutes 5 times per a week or stationery cycling for the same period. Executive function was measured using the Stroop C test, Colour Trials difference and The Digit Span Backwards test. Intention to treat analysis showed a significant group by time interaction in favour of the intervention group for the composite score on executive function (p = 0.002). In addition, group by time interaction were significant for all 3 measures of executive function. This was interesting as there were no differences between groups with regards to exercise frequency, intensity or duration. Cybercylist also decreased their risk for mild cognitive impairment by 23% when compared to traditional cyclist [19]. The post intervention results regarding executive function in this study are similar to our study, however, the Stroop task in both studies were the only common outcome measure. These cognitive effects could be due to the added mental effort required when participating in cybercycling compared to traditional cycling as both groups exerted similar physical efforts. The added mental effort such as competing with other riders, anticipating turns and dividing attention and decision making could partly tap on the domain of executive function.

The above mentioned studies have drawn similar conclusions to our study indicating that interactive video gaming does have the potential to improve executive function in ways similar to traditional moderate intensity exercise. One of the reasons for this could be due to the added mental effort required such as remembering sequences or instructions of a particular game when participating in interactive video gaming, in addition to the physical exertion.

In addition to significant improvements in executive function, our study also demonstrated that interactive video gaming significantly improved global cognitive function measured using the Mini-Mental State Examination over 12 weeks compared to the conventional multimodal supervised exercise group. The Mini-Mental State Examination has been widely used as a screening tool for dementia [29]. It has also been used frequently in research to measure change in cognition over time [42, 43]. A study by Suzuki and colleagues 2013 demonstrated the effects of 6 months of multicomponent exercise on memory function and general cognitive function in older adults with mild cognitive impairment [42]. Participants in the multicomponent exercise program participated in 90 minutes of exercise twice a week. The control group attended educational classes regarding health three times a week for the duration of the study. The Mini-Mental State Examination (p<0.04), logical memory (p<0.04) and reduced whole brain cortical atrophy (p<0.05) improved significantly in the multicomponent exercise group [42]. Similarly, Kwak et al.2008 showed the effects of exercise on cognitive function in 30 females with senile dementia [43]. Individuals in the exercise group participated in stretching, walking and upper and lower extremity exercises 2–3 times a week for 12 months with each session lasting between 30–60 minutes. After 12 months, the exercise group improved significantly (p<0.01) on the Mini-Mental State Examination compared to the control group [43]. Although Kwak et al.2008 found similar results to our study on the Mini-Mental State Examination, participants in their study were more impaired than our participants with evidence of memory complaints. However, contrasting results to the above-mentioned studies have also been observed.

Christofoletti et al. 2008 conducted a study whereby 54 individuals with mixed dementia (more than one type of dementia occurring simultaneously) were allocated into one of three groups namely: 1) An interdisciplinary therapy and education program; 2) a physiotherapy program; and 3) a non-active control group [44]. Groups 1 and 2 completed the intervention 5 and 3 times a week respectively for 6 months. Global cognitive function was measured using the Mini-Mental State Examination and The Brief Cognitive Screening Battery at baseline and post intervention. Global cognitive function did not improve through treatment however, attenuation was observed in the decline of global cognition on two specific domains in The Brief Cognitive Screening Battery for the interdisciplinary therapy group. The results indicate that exercise may slow the dementia related decline in cognitive symptoms, but lack statistical significance.

Our study also showed significant improvements in functional ability. To our knowledge, few studies have shown improvements in cardiorespiratory measures such as the 6 minute walk test in individuals participating in interactive video gaming. A study by Lund and Jessen. 2014 showed a significant improvement in the 6 minute walk test after 12 weeks using interactive modular stepping tiles as an intervention program [45]. The modular stepping tiles connect together to form a pressure-sensitive area of varying size and shape that allow the individuals to step on during the game and thereby providing feedback of the individuals movement. Individuals in this study participated in 9 sessions (1–1.5 hours per session) of modular interactive tiles over 12 weeks. The intervention group had a mean difference improvement of 22.4 percent when pre and post test scores were compared. Although an improvement was seen in the study conducted by Lund and Jessen.2014, there was no control group for comparison [45].

One of the possible reasons for improvement in the 6 minute walk test scores in our study could be due to the intensity and movement during each game compared to the comparison group’s conventional multimodal standing and seated exercise. The interactive gaming package used for our study requires many fitness components such as flexibility, agility, balance, lower body strength and endurance. Although our study did not measure intensity of the active gaming, a previous study by Graves et al. 2010 using Nintendo Wii Fit concluded that active gaming stimulated a light (Wii yoga, muscle conditioning and balance games) to moderate (Wii aerobics) intensity activity for young and older adults [46]. Therefore, the interactive video gaming sessions over 12 weeks could possibly have had a significant impact on individual’s cardiorespiratory fitness.

Our study showed that balance and mobility improved evidently by significant improvements for the intervention group in the functional reach, dynamic balance and timed up and go test. The interactive gaming group had significantly better scores on all of the above mentioned proxy measures of balance and mobility when compared to the conventional multimodal supervised exercise comparison group after 12 weeks. Previous studies have identified interactive video gaming as a means to improve balance and mobility in older adults [47, 48]. Bieryla and Dold. 2013 illustrated significant improvements in balance for individuals participating in Nintendo Wii Fit 3 times a week for 3 weeks compared to the control group who continued with normal activities. This is particularly important as an individual’s balance is coordinated by different physiological systems within the body. If this system is diminished, there is known to be an increased risk of falling in older adults [49]. In addition, falls have severe consequences that are accompanied by pain and decreased mobility for daily tasks such as walking up and down stairs. An improvement in balance may help reduce the risk of falling and thus indirectly improve an individual’s quality of life.

Similarly, Chien-Hung et al. 2013 also found positive effects of interactive video gaming on balance in older adults [48]. Group A participated in interactive video gaming for 6 weeks followed by no gaming for the next 6 weeks and Group B received no intervention for the first 6 weeks followed by gaming for the next 6 weeks. 12 weeks post intervention both groups improved significantly in balance measured by the Berg balance scale, Modified falls efficacy scale and the Timed up and Go test [48]. Our study found similar results to the above mentioned studies however; our study consisted of participants with some level of memory complaints while the previously mentioned studies excluded individuals with any cognitive impairment therefore including individuals with higher cognitive function. We must acknowledge that, although our study illustrated significant differences in balance between both groups in favour of the IVG group, the type of activities performed by both groups varied in nature. The intervention group performed active games in a standing position for most of their session (unless on a break in pairs) and therefore participated in more weight bearing compared to the comparison groups combination of standing and seated exercise. Participation in activities that are more weight bearing inclined may have a positive influence on balance.

In the present study, one of the main limitations was that the retirement homes were cluster-randomized into either the intervention or control group, rather than each individual being randomized into a group. The study was conducted in a single blind manner, although efforts were made to decrease bias on re-assessment. In addition, the version of the Stroop task used in this study did not include congruent words and therefore limited our analysis.

In summary, a low to moderate intensity interactive video gaming program has been shown to be effective in improving executive function and global cognitive function in older adults with subjective memory complaints. Furthermore, this intervention resulted in greater improvements in cognitive function than conventional multimodal supervised exercise. Interactive video gaming has also shown to be effective in improving functional reach, timed up and go and dynamic balance over 12 weeks which are proxy measures for balance and mobility. In addition, low to moderate intensity interactive video gaming has the potential to increase physical activity in older adults as it may overcome many physical activity barriers for older adults such as built up suburbs and cities that may not be suitable for activities such as walking and cycling, inclement weather and social isolation. Interventions of this nature will be sustainable and beneficial to older adults should volunteer workers be trained to conduct sessions at retirement homes and community centres. Further investigations on the long term effects of interactive video gaming in individuals with subjective memory complaints should be conducted with a larger sample size and a longer intervention period. This may be able to identify the optimal prescription of interactive video gaming for individuals with subjective memory complaints in the future.

## Supporting information

S1 Data Complete raw data pdf. (PDF)

S2 Data Complete raw data spreadsheet. (XLSX)

S1 Questionnaire Demographic and health status questionnaire. (PDF)
