# Supplementary material for: Efficacy of interactive video gaming in older adults with memory complaints: A cluster-randomized exercise intervention
Source: PLoS One. 2021 May 25;16(5):e0252016. doi: 10.1371/journal.pone.0252016 (PMC8148311; doi:10.1371/journal.pone.0252016)
Supplement: S1 Data — (PDF) [file pone.0252016.s001.pdf]

CODE	GROUP	CLUSTER	AGE	Heighta	Heightb	Weighta	Weightb	B.M.Ia
INT0001	1	1	76	1,64	1,64	69,6	67,8	25,87745
INT0002	1	1	77	1,58	1,57	88,3	89,4	35,37093
INT0003	1	1	74	1,5	1,51	57,8	56,8	25,68889
INT0004	1	1	71	1,54	1,53	65,2	62,5	27,49199
INT0005	1	1	66	1,62	1,62	83,6	83,9	31,8549
INT0007	1	1	71	1,62	1,62	100,1	101,4	38,14205
INT0008	1	2	73	1,6	1,61	85,3	85,9	33,32031
INT0009	1	1	67	1,56	1,56	59,8	60,6	24,57265
INT00010	1	2	81	1,51	1,51	53,9	52,3	23,63931
INT00011	1	2	76	1,65	1,7	89	86,8	32,69054
INT00012	1	2	66	1,64	1,63	72,2	74,3	26,84414
INT00013	1	1	68	1,62	1,62	73,5	71,5	28,0064
INT00014	1	1	65	1,6	1,59	65,4	65,4	25,54688
INT00015	1	1	66	1,59	1,58	60,8	59,7	24,04968
INT00016	1	1	76	1,55	1,56	65,3	68,1	27,18002
INT00017	1	3	71	1,53	1,52	64,3	60	27,46807
INT00018	1	3	68	1,61	1,6	69	69,5	26,61934
INT00020	1	3	72	1,53	1,53	56,8	55,8	24,26417
INT00021	1	3	66	1,62	1,6	115,8	115,5	44,12437
INT00023	1	3	72	1,49	1,48	65,7	64,3	29,59326
INT00024	1	3	74	1,51	1,5	65,7	65,3	28,81453
INT00026	1	3	65	1,58	1,57	87,1	87,1	34,89024
INT00027	1	3	68	1,57	1,57	48,9	48,8	19,83853
CTRL00041	2	4	71	1,52	1,52	55,6	59,2	24,0651
CTRL00042	2	4	71	1,52	1,52	64,4	62,1	27,87396
CTRL00043	2	4	75	1,63	1,63	61,1	59,4	22,99673
CTRL00045	2	4	65	1,59	1,58	107,6	104,4	42,56161
CTRL00047	2	4	83	1,61	1,61	71,9	71,1	27,73813
CTRL00048	2	5	80	1,7	1,7	71,3	71,2	24,67128
CTRL00049	2	5	72	1,62	1,62	76	73,3	28,959
CTRL00050	2	5	85	1,67	1,67	65,4	66	23,45011
CTRL00051	2	5	68	1,65	1,65	72,6	73,4	26,66667
CTRL00052	2	5	77	1,62	1,62	60,9	62	23,2053
CTRL00053	2	6	77	1,53	1,53	63	63	26,91273
CTRL00054	2	6	66	1,57	1,57	83,8	85,3	33,99732
CTRL00055	2	5	72	1,51	1,51	100,1	98,7	43,90158
CTRL00056	2	5	72	1,61	1,61	81,8	80,7	31,55742
CTRL00057	2	6	78	1,6	1,6	82,8	83,8	32,34375
CTRL00058	2	6	75	1,64	1,63	69,5	69,2	25,84027
CTRL00059	2	6	70	1,57	1,57	74,4	75	30,18378
CTRL00061	2	5	81	1,57	1,57	65,8	64,3	26,69479
CTRL00062	2	6	79	1,49	1,5	51,1	50,1	23,01698
CTRL00063	2	5	70	1,47	1,46	60,9	61,9	28,1827
CTRL00064	2	5	79	1,48	1,48	61,6	61,7	28,12272
CTRL00065	2	6	65	1,6	1,6	98,9	94,3	38,63281

B.M.Ib	Waist Circ.a	Waist Circ.b	Sub Scap.a	Sub Scap.b	Supra Iliac.a	Supra Iliac.b	Tricep a
25,20821	90	84,4	23	26	24	18	20
36,26922	105,5	97,5	37	35	29	28	31
24,91119	78	78	18	19	15	12	18
26,69913	87,5	84	24	21	22	18	20
31,96921	101,5	97	35	33	35	33	30
38,6374	103	97,5	35	38	31	31	34
33,13915	100,5	94,9	25	24	27	22	21
24,90138	86	82,5	26	23	26	28	20
22,93759	81,5	75,5	15	14	14	11	16
30,0346	99,5	95,6	24	29	30	22	22
27,96492	91	89,5	23	20	31	23	19
27,24432	90	84,5	27	24	20	16	18
25,86923	89	85,5	25	21	21	19	24
23,91444	80,4	77	18	18	15	10	11
27,98323	87	88	21	22	23	20	20
25,96953	74,8	68,8	22	19	28	12	23
27,14844	89,5	87	27	25	28	27	16
23,83699	72,5	69,4	22	17	18	16	23
45,11719	116,5	110,8	35	35	34	30	25
29,35537	84,5	81,4	31	28	18	17	28
29,02222	84,5	83	29	29	28	29	24
35,33612	94,5	91,8	31	30	24	24	26
19,79796	67,2	67,2	17	17	10	8	15
25,62327	67	69	20	20	15	14	21
26,87846	94,4	86,2	27	25	22	20	21
22,35688	72,5	71,5	13	14	15	11	15
41,82022	115	109,5	37	38	39	38	29
27,4295	101,5	97,6	33	29	32	30	20
24,63668	88,5	86	25	24	15	13	11
27,93019	94,5	91,8	33	34	34	30	11
23,66524	91,4	91	26	23	20	15	13
26,96051	85,5	85,4	29	32	24	20	15
23,62445	81	81,5	29	29	18	24	11
26,91273	77	77,8	23	27	18	17	18
34,60587	101	101,2	34	32	28	26	33
43,28758	103,3	101,5	43	38	25	29	32
31,13306	97	96	30	29	22	20	13
32,73438	79,5	82,9	21	25	22	23	30
26,04539	87,4	87,1	24	24	20	21	17
30,4272	87,2	89	27	26	21	20	17
26,08625	84	81,8	14	24	25	19	16
22,26667	71	70	20	23	19	18	16
29,03922	79,5	81,6	28	27	27	25	19
28,16837	76,5	77,1	21	25	15	17	18
36,83594	104,6	99,5	39	34	26	26	28

Tricep b	Bicep a	Bicep b	Tot. Skinfld a	Tot. Skinfld b	Body Density a	Body Density b	BF% a
17	11	8	78	69	1,02134204	1,024696507	34,65644
31	21	20	118	114	1,009609434	1,010552994	40,2886
17	8	7	59	55	1,029792323	1,031713151	30,67944
18	7	6	73	63	1,02518466	1,029215545	32,83984
27	20	21	120	114	1,013615581	1,015018994	38,35082
29	21	23	121	121	1,011358522	1,010952522	39,44068
25	15	14	88	85	1,019259592	1,019802608	35,64664
14	9	9	81	74	1,023963444	1,026030402	33,4157
11	5	6	50	42	1,03147889	1,036249295	29,89349
18	16	16	92	85	1,016825367	1,018990608	36,80926
17	10	9	83	69	1,02370208	1,028350507	33,53912
16	12	10	77	66	1,024943084	1,028754732	32,95365
23	11	9	81	72	1,024775444	1,027998053	33,03265
8	7	7	51	43	1,037027079	1,041695487	27,32601
19	12	15	76	76	1,022052744	1,022052744	34,31943
20	12	6	85	57	1,021020608	1,031953884	34,80902
16	15	12	86	80	1,021918598	1,023897331	34,383
20	15	13	78	66	1,02296604	1,027536732	33,88703
26	19	19	113	110	1,015260058	1,016402261	37,55981
24	17	12	94	81	1,017860945	1,021933444	36,31397
26	16	15	97	99	1,016189381	1,015630983	37,11393
26	19	19	100	99	1,01901	1,018878983	35,7656
13	6	5	48	43	1,037873802	1,040883487	26,9366
20	11	12	67	66	1,027531287	1,027942732	31,73716
19	7	12	77	76	1,023725084	1,024082744	33,52825
14	7	7	50	46	1,03391489	1,036196257	28,76281
35	20	21	125	132	1,012904669	1,011413842	38,69357
17	11	11	96	87	1,012818912	1,015512287	38,73495
8	8	7	59	52	1,027356323	1,030405789	31,8192
10	9	8	87	82	1,019978287	1,021597727	35,30445
12	10	8	69	58	1,021042507	1,025388036	34,79862
12	11	11	79	75	1,024241493	1,02566314	33,28446
13	9	10	67	76	1,025095287	1,021240744	32,88194
20	15	13	74	77	1,022376402	1,020883084	34,1661
36	26	28	121	122	1,013388522	1,012757331	38,46024
30	10	13	110	110	1,013560261	1,012748261	38,37747
11	12	11	77	71	1,023319084	1,025538724	33,72009
34	15	18	88	100	1,017229592	1,013732	36,61581
17	11	10	72	72	1,023938053	1,023938053	33,42768
18	11	13	76	77	1,024488744	1,024131084	33,16783
19	9	8	64	70	1,024724662	1,021866823	33,05659
17	8	8	63	66	1,025967545	1,024288732	32,4714
19	10	13	84	84	1,021750405	1,021750405	34,46274
17	11	12	65	71	1,025112459	1,022696724	32,87385
32	18	16	111	108	1,016154652	1,016904303	37,13057

BF% b	6 Min Walka	6 Min Walkb	Dynamic 6 Step a	Dynamic 6 Step b	Dynamic 10Ma
33,06986	537,5	565,5	9,1	3,9	45,9
39,83082	387,5	420	5,4	4,9	30,1
29,78452	437,5	524,5	6	4,7	36,6
30,94882	422,5	467,5	7,4	5,1	59,5
37,6756	431,2	470,6	6,3	8,1	39,6
39,63724	509,5	526	6,9	8	54,9
35,38805	367,5	434,5	7,8	7,5	66,7
32,44184	522	566,1	7,9	5	47,1
27,68428	524,4	541	9,1	5,4	63,5
35,77484	394,5	422,5	14,2	9,7	65,8
31,35339	500,25	537,6	3,8	3,8	31,5
31,16425	503,55	541,1	8,2	4	52
31,51842	438,5	565,5	9,9	5,9	48,7
25,18685	457,5	636,2	9,1	5	38,4
34,31943	420	403,8	8,3	7,1	41,9
29,6726	417,5	507,8	5	4,2	34
33,44691	515	664,1	9	5,9	64
31,7346	480	550,6	8,1	6,7	51,8
37,0119	443	420	5,8	5,7	22,5
34,37597	537,5	569,1	6,7	2,8	38,5
37,38174	454,5	487,5	6,1	3,7	40
35,82806	548,5	542,6	6,8	4,2	38,6
25,55755	525,5	567,6	6,7	6,3	37,3
31,54434	515,5	499	5,1	6,5	36,2
33,35938	323,5	351,5	10	7,2	43,3
27,70873	595,5	600,5	5,2	5,6	32,3
39,41391	338,5	397,6	6,5	6,1	50
37,43871	445,5	430	10,6	11,8	71,6
30,39326	454,5	492,5	9,6	7,7	41,7
34,53514	590,5	612,6	3,9	4,1	22,5
32,74408	427,6	406	7	6,7	51,3
32,61459	630	654,4	8,8	6,1	48,6
34,70452	623,8	647,5	5,4	6,9	38,1
34,87433	538,2	522,4	7,7	10,5	55
38,76467	475	478,7	12,5	9,5	88,2
38,76904	295	284,8	6,7	10,6	48,1
32,67314	424,5	502,6	5,7	4,6	32,6
38,29474	515,1	525,1	8,5	10,4	43,6
33,42768	560	574,9	8,9	6,1	66,9
33,33656	551,5	602,4	4,5	5	25
34,40755	485,5	507,7	9,1	14,7	56,6
33,26217	530	505	6,1	6,4	43,3
34,46274	437,6	469,8	8,1	6,5	56,8
34,01446	437,5	438,6	8	6,4	54,4
36,77147	584,8	547,4	7,7	6,6	36,9

Dynamic 10Mb	Timed Up &	Timed Up &	Grip Left 1a	Grip Left 2a	Grip Left 1b	Grip Left 2b	Grip Left Av
28,3	5,83	5,9	22,9	21,8	21,8	24,3	22,35
24,4	7,9	6,5	12,4	11,4	15,1	14,7	11,9
33,8	6,53	5,9	20,3	17,1	20,1	18	18,7
42,3	7,85	6,4	20,4	20,1	20,4	20,1	20,25
50,3	7,4	6,7	25,1	22,9	24	21,2	24
38,8	7,4	6,2	27,2	24	26,4	23,2	25,6
45,7	8	6,4	18	18	20,4	19,6	18
31,1	5,9	5,7	16	16	16,6	16,1	16
37	7	5,8	17,6	19,9	16	18,7	18,75
46,2	9,8	8,1	22,5	20,5	22,8	21,7	21,5
29,2	6,8	6	18,4	20	19,3	21,1	19,2
29,1	7	7	23,9	22,8	21,8	21,8	23,35
43,7	6	6,4	23,9	25,7	24,2	23,7	24,8
30,8	7,5	6,3	35,1	31	30,7	32,4	33,05
41,7	8,5	7,3	15,7	13	16,2	11,4	14,35
27,4	8,7	5,8	19,3	19,4	18,6	16	19,35
37,1	7,1	4,9	26,7	26,7	25,8	26,7	26,7
39,8	7	5,9	16,9	15	16,4	15	15,95
26,6	6,6	5,3	25,4	25,6	27,2	27,1	25,5
21,6	7,4	5,2	13,1	15,3	13,4	16,1	14,2
28,8	8,7	6	17,4	15,6	17,4	16,6	16,5
26,6	6,6	6	19,3	18,7	19,7	15,6	19
45,4	6,3	5,4	19,9	19	20,8	20,8	19,45
42,8	8,7	6,5	20,1	23,8	13,4	19,1	21,95
43,3	12,4	12,8	16,6	17,6	18,7	17,4	17,1
30,6	6,9	5,9	20,3	20,3	22,9	21,1	20,3
37,3	6,8	6,5	23,2	18,1	13,1	15,4	20,65
48,9	9,9	9,3	14	13,9	10,6	10,9	13,95
43,2	8,2	7,6	35,4	34,4	36,4	35,3	34,9
21,3	5,3	5,9	32,6	31,9	31,4	28,3	32,25
50,4	7,9	7,1	24,2	24,2	24,7	24	24,2
33,7	5,3	5,7	27,6	27,6	30,1	30,3	27,6
39,6	6,1	6,9	15	16,7	12,3	15,4	15,85
66,7	6,1	7,4	24,7	22,6	21,9	20,3	23,65
69,2	6,6	6,8	17,1	17,4	17,4	17	17,25
62,2	9,7	9,3	13,1	12,3	10,4	11,3	12,7
28,2	7,4	6,8	34	25,7	27,9	25,3	29,85
58,8	6,5	8,8	18,4	18,7	20,1	20,7	18,55
40	6,6	8,7	28,2	30,7	26,7	25,4	29,45
32,2	5,3	5,1	28,3	30,7	31,8	34,4	29,5
70,7	6,9	8	18,6	20,3	18,1	18,1	19,45
39,8	6,5	7,4	18,1	18,3	17,4	17,5	18,2
45,8	7,3	7,6	17,9	15,1	15,3	15,7	16,5
38	6,1	7	13,5	13	14,9	14,1	13,25
34,9	7,1	7,6	21,3	18,6	24,7	24,6	19,95

Grip Left A	Grip Right :	Grip Right :	Grip Right :	Grip Right :	Grip Right /	Grip Right /	F.R 1a	F.R 1b
23,05	22,2	26,6	24,2	27,2	24,4	25,7	75	90,1
14,9	11,6	13,1	14,7	14,7	12,35	14,7	79	84,1
19,05	22,8	20,4	21,4	19,1	21,6	20,25	77	80
20,25	19,7	19,7	19,7	19,7	19,7	19,7	75,5	81
22,6	23,9	24,4	22,6	24,7	24,15	23,65	80	82,6
24,8	22,9	27,6	25,3	26,9	25,25	26,1	90	92,5
20	14,9	14,1	21,3	18,6	14,5	19,95	76,5	83,7
16,35	15,4	13,9	16,3	16,4	14,65	16,35	74	82,2
17,35	19,4	21	18,7	17,9	20,2	18,3	82	89,8
22,25	20,3	21,3	23,3	22,4	20,8	22,85	79,5	88,9
20,2	13,7	15	17,4	15,3	14,35	16,35	96,5	94,4
21,8	29,4	28,1	26,5	24,3	28,75	25,4	77,5	88,7
23,95	24,2	21,9	24,3	23,5	23,05	23,9	88,4	91
31,55	34,1	34,9	32,6	31,1	34,5	31,85	82,5	81,6
13,8	12,1	13,4	14,3	13,6	12,75	13,95	79	80,5
17,3	19,4	18,4	20,3	20,4	18,9	20,35	82,6	84,6
26,25	25,6	25,8	22,2	21,8	25,7	22	84	87,5
15,7	18,6	19,5	20,4	18	19,05	19,2	64,4	81
27,15	27,6	27,6	28,1	29,6	27,6	28,85	74,5	80,2
14,75	15,3	20,7	17,4	18,9	18	18,15	78,8	82,2
17	19,9	15,6	16,5	16,3	17,75	16,4	81,2	78,5
17,65	19	20,2	20,3	16,1	19,6	18,2	77,7	80
20,8	24,9	25,8	23,9	24	25,35	23,95	91,2	90,5
16,25	21,9	23,1	19,3	14	22,5	16,65	72,5	73,8
18,05	16,1	13,4	15,9	15,9	14,75	15,9	67,5	74,2
22	23,6	20,1	20,7	22,8	21,85	21,75	86,2	89,2
14,25	22,7	21,9	22,6	21,1	22,3	21,85	76	81,5
10,75	11,6	12,7	10,3	8,8	12,15	9,55	80,1	77,9
35,85	33,4	34,1	32	30,3	33,75	31,15	85,8	86,2
29,85	36,1	37,1	38,3	36,4	36,6	37,35	85,1	86,2
24,35	24,7	25,3	26,8	25	25	25,9	85,5	83,2
30,2	27,4	28,6	31,3	35	28	33,15	85	84,8
13,85	16	16,4	16,7	16,6	16,2	16,65	89,1	82,2
21,1	24,2	21,8	20,6	21,7	23	21,15	89,9	83,1
17,2	18,1	17,9	16,6	16,7	18	16,65	85	80,8
10,85	15,1	15,4	18,6	16,3	15,25	17,45	75,7	78,3
26,6	37,1	35,7	26,5	30,7	36,4	28,6	84,6	78
20,4	24,6	24,2	23,6	23,8	24,4	23,7	87,8	84
26,05	30,3	31,4	22,6	21,7	30,85	22,15	97	84,7
33,1	33,4	34,1	35,3	32,3	33,75	33,8	90,2	85,3
18,1	16,1	19,4	18,9	20,8	17,75	19,85	77,8	79,1
17,45	16,7	15,3	19	16	16	17,5	76,7	84,2
15,5	18,9	17,1	21,3	16,6	18	18,95	75,1	74,7
14,5	14,1	11,7	12,2	12,1	12,9	12,15	86,1	81,2
24,65	18,3	17,1	22,2	20,8	17,7	21,5	84,6	83

F.R 2a	F.R 2b	F.R 3a	F.R 3b	F.R Ave a	F.R Ave b	Ave Rt of Mistakes a
78	89,9	79,5	91	77,5	90,33333	2115
79,5	86,1	80	87,2	79,5	85,8	1214
79	83,4	78,5	82,5	78,16667	81,96667	1573
77	81,3	77,5	82,3	76,66667	81,53333	2013
78	83,1	79,5	93,2	79,16667	86,3	1708
90,5	93,9	91	94,3	90,5	93,56667	1450
75	83,7	77	83,2	76,16667	83,53333	2130
80	83,5	82,5	83,8	78,83333	83,16667	2054
82,8	89	84,3	89,4	83,03333	89,4	2112
78	87,7	77,2	89	78,23333	88,53333	1667
98	95,5	95,7	96	96,73333	95,3	0
74	88,5	76,4	89,1	75,96667	88,76667	2314
87,4	89,5	86,2	90,2	87,33333	90,23333	1487
81,4	82	83	83,4	82,3	82,33333	0
77,6	80,3	81,2	83,3	79,26667	81,36667	1855
82,5	85,1	84,8	87,2	83,3	85,63333	1567
82	86	82,8	88,4	82,93333	87,3	1819
65,4	79,8	66	78,8	65,26667	79,86667	1624
73,8	83,3	75,5	83,5	74,6	82,33333	1260
80	83,5	81,2	84,2	80	83,3	1284
83,5	79,5	83	80,2	82,56667	79,4	1574
80	83,4	83,6	84,5	80,43333	82,63333	1647
90,5	90,1	91	91,6	90,9	90,73333	0
74	76,4	78	74,5	74,83333	74,9	1412
70,7	73,5	71,5	74,4	69,9	74,03333	1571
88	89,5	88,5	91,4	87,56667	90,03333	1566
79,4	80	79,5	83,3	78,3	81,6	1202
80,1	78	81,3	81	80,5	78,96667	1082
87,5	87,3	88	90,2	87,1	87,9	2297
85	85,5	85,3	87,2	85,13333	86,3	1155
86,1	87,5	84,4	84,5	85,33333	85,06667	2034
83,2	83,2	81,5	82,3	83,23333	83,43333	1416
89,5	87	89,6	90,1	89,4	86,43333	1688
88,5	83,5	90,4	81,4	89,6	82,66667	1509
86,4	83,8	85,6	81,4	85,66667	82	0
76,1	78,3	76,2	80,2	76	78,93333	1873
83,8	76,2	84,5	78,2	84,3	77,46667	1235
89,7	85,9	86,4	87,8	87,96667	85,9	1239
94,6	85,6	94,9	83,6	95,5	84,63333	1194
90,5	81,1	90	83	90,23333	83,13333	2057
78	80,1	78,4	79	78,06667	79,4	1421
79,5	82,2	80,4	83	78,86667	83,13333	1792
75,2	74,4	74	73,5	74,76667	74,2	1749
84,1	83,5	81,5	86,8	83,9	83,83333	1650
85,5	84,6	85,8	84,1	85,3	83,9	1761

Ave Rt of Mistakes +1a	LOG Ave Rt of Mistakes +1a	Ave Rt of Mistakes b	Ave Rt of Mistakes +1b
2116	3,325515663	1865	1866
1215	3,084576278	1260	1261
1574	3,197004728	1833	1834
2014	3,304059466	1879	1880
1709	3,232742063	1453	1454
1451	3,161667412	1009	1010
2131	3,32858345	1321	1322
2055	3,312811826	0	1
2113	3,324899497	1994	1995
1668	3,222196046	1162	1163
1	0	1087	1088
2315	3,364550995	1616	1617
1488	3,172602931	0	1
1	0	1648	1649
1856	3,268577972	2368	2369
1568	3,195346058	1458	1459
1820	3,260071388	2026	2027
1625	3,210853365	1944	1945
1261	3,100715087	1556	1557
1285	3,108903128	1577	1578
1575	3,197280558	1900	1901
1648	3,216957207	0	1
1	0	0	1
1413	3,150142162	2071	2072
1572	3,196452542	2027	2028
1567	3,195068996	1520	1521
1203	3,080265627	0	1
1083	3,034628457	1510	1511
2298	3,361350024	2078	2079
1156	3,062957834	1126	1127
2035	3,308564414	1715	1716
1417	3,15136985	1558	1559
1689	3,22762965	1502	1503
1510	3,178976947	2166	2167
1	0	0	1
1874	3,272769587	2077	2078
1236	3,092018471	2245	2246
1240	3,093421685	0	1
1195	3,077367905	1822	1823
2058	3,31344537	1772	1773
1422	3,152899596	2698	2699
1793	3,25358029	1764	1765
1750	3,243038049	1961	1962
1651	3,217747073	1596	1597
1762	3,246005904	1918	1919

LOG Ave Rt of Mistakes +1b	Total Mistakes a	Total Mistakes b	% Total Mistakes a
3,270911639	6	13	18,75
3,100715087	9	11	28,125
3,263399331	9	13	28,125
3,274157849	9	7	28,125
3,162564407	6	3	18,75
3,004321374	10	1	31,25
3,121231455	6	1	18,75
0	10	0	31,25
3,2999429	5	6	15,625
3,065579715	6	5	18,75
3,036628895	0	1	0
3,20871002	4	0	12,5
0	2	0	6,25
3,217220656	0	1	0
3,374565061	14	11	43,75
3,164055292	11	2	34,375
3,306853749	5	1	15,625
3,288919606	22	8	68,75
3,192288613	3	0	9,375
3,198106999	8	4	25
3,278982117	9	4	28,125
0	4	0	12,5
0	0	0	0
3,316389751	18	6	56,25
3,307067951	6	6	18,75
3,182129214	2	1	6,25
0	4	0	12,5
3,179264464	6	2	18,75
3,317854489	8	3	25
3,051923916	4	2	12,5
3,234517284	9	11	28,125
3,192846115	1	4	3,125
3,176958981	9	5	28,125
3,335858911	6	5	18,75
0	0	0	0
3,317645543	2	9	6,25
3,351409752	9	13	28,125
0	16	0	50
3,260786669	7	8	21,875
3,248708736	1	2	3,125
3,431202885	1	1	3,125
3,24674471	14	7	43,75
3,292699003	11	3	34,375
3,203304916	11	19	34,375
3,283074975	10	15	31,25

% Total Mistakes b	Total Missed Resp. a	Total Missed Resp. b	Total Correct a	Total Correct b
40,625	12	3	14	16
34,375	2	0	21	21
40,625	11	5	12	14
21,875	8	2	15	23
9,375	9	2	17	27
3,125	6	0	16	31
3,125	3	0	23	31
0	1	0	21	32
18,75	5	2	22	24
15,625	1	0	25	27
3,125	1	0	31	31
0	2	0	26	32
0	0	0	30	32
3,125	0	0	32	31
34,375	8	8	10	13
6,25	1	0	20	30
3,125	7	1	20	30
25	1	3	9	21
0	1	0	28	32
12,5	1	1	23	27
12,5	4	2	19	26
0	0	0	28	32
0	0	1	32	31
18,75	2	11	12	15
18,75	10	9	16	17
3,125	0	0	30	31
0	1	0	27	32
6,25	3	0	23	30
9,375	3	24	21	5
6,25	2	0	26	30
34,375	12	6	11	15
12,5	0	0	31	28
15,625	4	3	19	24
15,625	12	9	14	18
0	0	0	32	32
28,125	3	2	27	21
40,625	9	5	14	14
0	0	0	16	32
25	0	0	25	24
6,25	2	1	29	29
3,125	0	0	31	31
21,875	2	0	16	25
9,375	2	3	19	26
59,375	4	1	17	12
46,875	1	1	21	16

% Total Correct a	LOG % Total Correc	% Total Correct b	LOG % Total Correc
43,75	1,640978057	50	1,698970004
65,625	1,817069316	65,625	1,817069316
37,5	1,574031268	43,75	1,640978057
46,875	1,670941281	71,875	1,856577858
53,125	1,725298943	84,375	1,926213786
50	1,698970004	96,875	1,986211716
71,875	1,856577858	96,875	1,986211716
65,625	1,817069316	100	2
68,75	1,837272703	75	1,875061263
78,125	1,89279003	84,375	1,926213786
96,875	1,986211716	96,875	1,986211716
81,25	1,90982337	100	2
93,75	1,971971276	100	2
100	2	96,875	1,986211716
31,25	1,494850022	40,625	1,608793374
62,5	1,795880017	93,75	1,971971276
62,5	1,795880017	93,75	1,971971276
28,125	1,449092531	65,625	1,817069316
87,5	1,942008053	100	2
71,875	1,856577858	84,375	1,926213786
59,375	1,773603623	81,25	1,90982337
87,5	1,942008053	100	2
100	2	96,875	1,986211716
37,5	1,574031268	46,875	1,670941281
50	1,698970004	53,125	1,725298943
93,75	1,971971276	96,875	1,986211716
84,375	1,926213786	100	2
71,875	1,856577858	93,75	1,971971276
65,625	1,817069316	15,625	1,193820026
81,25	1,90982337	93,75	1,971971276
34,375	1,536242707	46,875	1,670941281
96,875	1,986211716	87,5	1,942008053
59,375	1,773603623	75	1,875061263
43,75	1,640978057	56,25	1,750122527
100	2	100	2
84,375	1,926213786	65,625	1,817069316
43,75	1,640978057	43,75	1,640978057
50	1,698970004	100	2
78,125	1,89279003	75	1,875061263
90,625	1,95724802	90,625	1,95724802
96,875	1,986211716	96,875	1,986211716
50	1,698970004	78,125	1,89279003
59,375	1,773603623	81,25	1,90982337
53,125	1,725298943	37,5	1,574031268
65,625	1,817069316	50	1,698970004

Ave Rt of All Correct Resp. a	LOG Ave Rt of All Correct Resp. a	Ave Rt of All Correct Resp. b
2125	3,327358934	1789
1844	3,265760917	1655
1965	3,293362555	1815
2038	3,30920418	1722
1622	3,21005085	1462
1370	3,136720567	1354
1900	3,278753601	1588
1467	3,166430114	1559
2021	3,305566314	1768
1566	3,194791758	1422
1691	3,228143608	1198
1914	3,281941933	1541
1583	3,199480915	1457
1589	3,201123897	1663
1763	3,246252312	2190
1638	3,214313897	1385
1514	3,180125875	1407
1565	3,194514342	1804
1351	3,130655349	1295
1448	3,160768562	1244
1745	3,241795431	1862
1689	3,22762965	1548
1265	3,102090526	1247
1686	3,22685757	1571
1619	3,209246849	1925
1614	3,20790353	1352
1602	3,204662512	1456
2100	3,322219295	1719
1809	3,257438567	1904
1663	3,220892249	1375
2140	3,330413773	2284
1609	3,206556044	1559
1379	3,139564266	1585
1999	3,300812794	1831
1387	3,142076461	1463
1659	3,219846386	1880
1113	3,046495164	2172
1422	3,152899596	1616
1333	3,124830149	1580
1652	3,218010043	1332
1585	3,200029267	1696
2002	3,301464073	1598
1932	3,286007122	1895
1778	3,249931757	1524
1729	3,237794993	1912

LOG Ave Rt of All Correct Resp. b	Grey Mistakes.a	Grey Mistakes.b	Grey Missed.a
3,252610341	1	7	4
3,218797998	2	3	1
3,258876629	0	4	8
3,236033147	2	1	3
3,164947373	1	1	7
3,131618664	2	1	3
3,200850498	0	0	1
3,192846115	4	0	1
3,247482261	0	2	2
3,152899596	1	0	1
3,078456818	0	0	0
3,187802639	0	0	0
3,163459552	2	0	0
3,220892249	0	0	0
3,340444115	1	3	0
3,141449773	2	0	1
3,148294097	0	1	5
3,256236533	1	6	0
3,112269768	0	0	1
3,09482038	2	1	1
3,269979677	4	0	3
3,189770956	0	0	0
3,095866453	0	0	0
3,196176185	3	0	0
3,284430734	0	2	4
3,130976692	1	0	0
3,163161375	2	0	0
3,235275877	2	1	1
3,279666944	3	0	2
3,138302698	3	1	1
3,3586961	0	2	7
3,192846115	0	0	0
3,200029267	3	4	2
3,262688344	1	1	7
3,165244326	0	0	0
3,274157849	0	1	2
3,336859821	0	1	8
3,208441356	6	0	0
3,198657087	6	0	0
3,124504225	0	0	0
3,229425848	0	0	0
3,203576775	1	3	1
3,277609214	0	0	1
3,182984967	4	2	2
3,281487888	0	1	1

Grey Missed.b	Grey Correct.a	Grey Correct.b	% of Correct Grey.a	% of Correct Grey. A +1
0	3	1	37,5	38,5
0	5	5	62,5	63,5
1	0	3	0	1
1	3	6	37,5	38,5
0	0	7	0	1
0	3	7	37,5	38,5
0	7	8	87,5	88,5
0	3	8	37,5	38,5
2	6	4	75	76
0	6	8	75	76
0	8	8	100	101
0	8	8	100	101
0	6	8	75	76
0	8	8	100	101
2	7	3	87,5	88,5
0	5	8	62,5	63,5
0	3	7	37,5	38,5
0	7	2	87,5	88,5
0	7	8	87,5	88,5
1	5	6	62,5	63,5
2	1	6	12,5	13,5
0	8	8	100	101
0	8	8	100	101
3	5	5	62,5	63,5
2	4	4	50	51
0	7	8	87,5	88,5
0	6	8	75	76
0	5	7	62,5	63,5
8	3	0	37,5	38,5
0	4	7	50	51
2	1	4	12,5	13,5
0	8	8	100	101
1	3	3	37,5	38,5
5	0	2	0	1
0	8	8	100	101
0	6	7	75	76
3	0	4	0	1
0	2	8	25	26
0	2	8	25	26
1	8	7	100	101
0	8	8	100	101
0	6	5	75	76
0	7	8	87,5	88,5
0	2	6	25	26
0	7	7	87,5	88,5

LOG % of Correct Grey. A +1	% of Correct Grey.b	% of Correct Grey.b +1
1,58546073	12,5	13,5
1,802773725	62,5	63,5
0	37,5	38,5
1,58546073	75	76
0	87,5	88,5
1,58546073	87,5	88,5
1,946943271	100	101
1,58546073	100	101
1,880813592	50	51
1,880813592	100	101
2,004321374	100	101
2,004321374	100	101
1,880813592	100	101
2,004321374	100	101
1,946943271	37,5	38,5
1,802773725	100	101
1,58546073	87,5	88,5
1,946943271	25	26
1,946943271	100	101
1,802773725	75	76
1,130333768	75	76
2,004321374	100	101
2,004321374	100	101
1,802773725	62,5	63,5
1,707570176	50	51
1,946943271	100	101
1,880813592	100	101
1,802773725	87,5	88,5
1,58546073	0	1
1,707570176	87,5	88,5
1,130333768	50	51
2,004321374	100	101
1,58546073	37,5	38,5
0	25	26
2,004321374	100	101
1,880813592	87,5	88,5
0	50	51
1,414973348	100	101
1,414973348	100	101
2,004321374	87,5	88,5
2,004321374	100	101
1,880813592	62,5	63,5
1,946943271	100	101
1,414973348	75	76
1,946943271	87,5	88,5

LOG % of Correct Grey. b +1	Ave Rt of Correct Grey (NEUTRAL) Words.a
1,130333768	1793
1,802773725	2236
1,58546073	0
1,880813592	1657
1,946943271	0
1,946943271	1385
2,004321374	2187
2,004321374	2173
1,707570176	2334
2,004321374	1827
2,004321374	1877
2,004321374	2096
2,004321374	2034
2,004321374	1729
1,58546073	1678
2,004321374	1697
1,946943271	1575
1,414973348	1536
2,004321374	1678
1,880813592	1880
1,880813592	2603
2,004321374	1725
2,004321374	1238
1,802773725	1803
1,707570176	1853
2,004321374	1752
2,004321374	1728
1,946943271	2288
0	2080
1,946943271	2356
1,707570176	1464
2,004321374	1753
1,58546073	1792
1,414973348	0
2,004321374	1557
1,946943271	2098
1,707570176	0
2,004321374	1447
2,004321374	1159
1,946943271	2155
2,004321374	1688
1,802773725	2140
2,004321374	1854
1,880813592	2823
1,946943271	2127

Ave Rt of Correct Grey (NEUTRAL) Words.a +1	LOG Ave Rt of Correct Grey (NEUTRAL) Words.a +1
1794	3,253822439
2237	3,349665984
1	0
1658	3,219584526
1	0
1386	3,14176323
2188	3,340047318
2174	3,33725954
2335	3,368286885
1828	3,261976191
1878	3,273695588
2097	3,32159843
2035	3,308564414
1730	3,238046103
1679	3,225050696
1698	3,229937686
1576	3,197556213
1537	3,186673867
1679	3,225050696
1881	3,274388796
2604	3,41564098
1726	3,237040791
1239	3,093071306
1804	3,256236533
1854	3,26810973
1753	3,243781916
1729	3,237794993
2289	3,359645793
2081	3,31827208
2357	3,372359583
1465	3,165837625
1754	3,244029589
1793	3,25358029
1	0
1558	3,192567453
2099	3,322012439
1	0
1448	3,160768562
1160	3,064457989
2156	3,333648757
1689	3,22762965
2141	3,330616667
1855	3,268343914
2824	3,450864692
2128	3,327971624

Ave Rt of Correct Grey (NEUTRAL) Words.b	Ave Rt of Correct Grey (NEUTRAL) Words.b +1
2087	2088
2101	2102
2456	2457
2140	2141
1692	1693
1649	1650
1618	1619
1816	1817
1489	1490
1677	1678
1451	1452
1616	1617
1847	1848
1793	1794
2261	2262
1462	1463
1533	1534
1512	1513
1556	1557
1430	1431
2167	2168
1803	1804
1222	1223
1859	1860
1524	1525
1404	1405
1651	1652
1754	1755
0	1
1768	1769
2296	2297
1609	1610
2148	2149
2552	2553
1469	1470
2035	2036
2178	2179
1621	1622
1640	1641
1545	1546
1992	1993
1730	1731
1755	1756
1713	1714
2059	2060

LOG Ave Rt of Correct Grey (NEUTRAL) Words.b +1
3,319730494
3,322632712
3,390405156
3,330616667
3,228656958
3,217483944
3,209246849
3,259354927
3,173186268
3,224791956
3,161966616
3,20871002
3,266701967
3,253822439
3,354492601
3,165244326
3,18582536
3,179838928
3,192288613
3,155639634
3,336059278
3,256236533
3,087426457
3,269512944
3,183269844
3,147676324
3,218010043
3,244277121
0
3,247727833
3,361160995
3,206825876
3,332236415
3,407050815
3,167317335
3,308777774
3,33825723
3,21005085
3,215108581
3,18920949
3,299507299
3,238297068
3,244524512
3,234010818
3,31386722

No. of Correct Colour Word (INCONGRUENT) Responses.a
11
16
12
12
17
13
16
18
16
19
23
18
24
24
3
15
17
2
21
18
18
20
24
7
12
23
21
18
18
22
10
23
16
14
24
21
14
14
23
21
23
10
12
15
14

No. of Correct Colour Word (INCONGRUENT) Responses.b
15
16
11
17
20
24
23
24
20
19
23
24
24
23
10
22
23
19
24
21
20
24
23
10
13
23
24
23
5
23
11
20
21
16
24
14
10
24
16
22
23
20
18
6
9

% of Correct Colour Words (INCONGRUENT) a	LOG % of Correct Colour Words (INCONGRUENT) a
45,83333333	1,661181443
66,66666667	1,823908741
50	1,698970004
50	1,698970004
70,83333333	1,85023768
54,16666667	1,733732111
66,66666667	1,823908741
75	1,875061263
66,66666667	1,823908741
79,16666667	1,898542359
95,83333333	1,981516594
75	1,875061263
100	2
100	2
12,5	1,096910013
62,5	1,795880017
70,83333333	1,85023768
8,333333333	0,920818754
87,5	1,942008053
75	1,875061263
75	1,875061263
83,33333333	1,920818754
100	2
29,16666667	1,464886798
50	1,698970004
95,83333333	1,981516594
87,5	1,942008053
75	1,875061263
75	1,875061263
91,66666667	1,962211439
41,66666667	1,619788758
95,83333333	1,981516594
66,66666667	1,823908741
58,33333333	1,765916794
100	2
87,5	1,942008053
58,33333333	1,765916794
58,33333333	1,765916794
95,83333333	1,981516594
87,5	1,942008053
95,83333333	1,981516594
41,66666667	1,619788758
50	1,698970004
62,5	1,795880017
58,33333333	1,765916794

% of Correct Colour Words (INCONGRUENT) b
62,5
66,66666667
45,83333333
70,83333333
83,33333333
100
95,83333333
100
83,33333333
79,16666667
95,83333333
100
100
95,83333333
41,66666667
91,66666667
95,83333333
79,16666667
100
87,5
83,33333333
100
95,83333333
41,66666667
54,16666667
95,83333333
100
95,83333333
20,83333333
95,83333333
45,83333333
83,33333333
87,5
66,66666667
100
58,33333333
41,66666667
100
66,66666667
91,66666667
95,83333333
83,33333333
75
25
37,5

LOG % of Correct Colour Words (INCONGRUENT) b
1,795880017
1,823908741
1,661181443
1,85023768
1,920818754
2
1,981516594
2
1,920818754
1,898542359
1,981516594
2
2
1,981516594
1,619788758
1,962211439
1,981516594
1,898542359
2
1,942008053
1,920818754
2
1,981516594
1,619788758
1,733732111
1,981516594
2
1,981516594
1,318758763
1,981516594
1,661181443
1,920818754
1,942008053
1,823908741
2
1,765916794
1,619788758
2
1,823908741
1,962211439
1,981516594
1,920818754
1,875061263
1,397940009
1,574031268

Ave Rt of Correct Colour Word (INCONGRUENT) Responses a
2215
1721
1965
2134
1622
1366
1774
1349
1903
1484
1626
1833
1470
1542
1959
1618
1504
1668
1242
1329
1697
1675
1274
1603
1540
1572
1567
2048
1764
1537
2208
1558
1301
1999
1330
1534
1113
1419
1348
1461
1549
1919
1977
1639
1530

LOG Ave Rt of Correct Colour Word (INCONGRUENT) Responses a
3,345373731
3,23578087
3,293362555
3,329194415
3,21005085
3,135450699
3,248953615
3,13001195
3,279438788
3,171433901
3,211120541
3,263162465
3,167317335
3,188084374
3,292034436
3,208978517
3,177247836
3,222196046
3,094121596
3,123524981
3,229681842
3,224014811
3,105169428
3,204933522
3,187520721
3,196452542
3,195068996
3,311329952
3,246498581
3,186673867
3,343999069
3,192567453
3,114277297
3,300812794
3,123851641
3,18582536
3,046495164
3,151982395
3,129689892
3,164650216
3,190051418
3,283074975
3,296006669
3,214578954
3,184691431

Ave Rt of Correct Colour Word (INCONGRUENT) Responses b
1769
1516
1640
1574
1381
1267
1577
1474
1824
1315
1110
1517
1327
1618
2169
1357
1369
1835
1209
1191
1771
1463
1256
1426
2048
1334
1391
1709
1904
1256
2280
1538
1504
1741
1461
1802
2170
1614
1550
1264
1593
1565
1957
1335
1797

LOG Ave Rt of Correct Colour Word (INCONGRUENT) Responses b
3,247727833
3,180699201
3,214843848
3,197004728
3,140193679
3,102776615
3,197831693
3,168497484
3,261024834
3,118925753
3,045322979
3,180985581
3,122870923
3,208978517
3,336259552
3,132579848
3,136403448
3,263636069
3,082426301
3,075911761
3,248218561
3,165244326
3,098989639
3,154119526
3,311329952
3,12515583
3,14332713
3,232742063
3,279666944
3,098989639
3,357934847
3,186956335
3,177247836
3,240798771
3,164650216
3,255754787
3,336459734
3,20790353
3,190331698
3,101747074
3,202215776
3,194514342
3,291590826
3,125481266
3,254548077

Ave Rt of Incorrect Colour Word (INCONGRUENT) Responses a

2398
1223
1573
1787
1679
1289
2130
1753
2112
1786
0
2314
0
0
1853
1555
1819
1623
1260
1239
1492
1647
0
1475
1571
1848
1227
1287
2070
836
2034
1416
1648
1645
0
1873
1235
1207
1265
2057
1421
1750
1749
1762
1761

Ave Rt of Incorrect Colour Word (INCONGRUENT) Responses a +1
2399
1224
1574
1788
1680
1290
2131
1754
2113
1787
1
2315
1
1
1854
1556
1820
1624
1261
1240
1493
1648
1
1476
1572
1849
1228
1288
2071
837
2035
1417
1649
1646
1
1874
1236
1208
1266
2058
1422
1751
1750
1763
1762

LOG Ave Rt of Incorrect Colour Word (INCONGRUENT) Responses a +1
3,380030248
3,087781418
3,197004728
3,252367514
3,225309282
3,11058971
3,32858345
3,244029589
3,324899497
3,252124553
0
3,364550995
0
0
3,26810973
3,192009593
3,260071388
3,210586025
3,100715087
3,093421685
3,174059808
3,216957207
0
3,169086357
3,196452542
3,266936911
3,089198367
3,109915863
3,316180099
2,922725458
3,308564414
3,15136985
3,217220656
3,216429831
0
3,272769587
3,092018471
3,082066934
3,102433706
3,31344537
3,152899596
3,243286146
3,243038049
3,246252312
3,246005904

Ave Rt of Incorrect Colour Word (INCONGRUENT) Responses b
1597
1119
1781
1767
1545
0
1321
0
1874
1163
1087
0
0
1048
2488
1458
0
2134
0
1741
1900
0
0
2071
1773
1520
0
1587
2078
1322
1534
1558
1740
2203
0
2168
2266
0
1822
1772
2698
1626
1961
1600
1993

Ave Rt of Incorrect Colour Word (INCONGRUENT) Responses b +1
1598
1120
1782
1768
1546
1
1322
1
1875
1164
1088
1
1
1049
2489
1459
1
2135
1
1742
1901
1
1
2072
1774
1521
1
1588
2079
1323
1535
1559
1741
2204
1
2169
2267
1
1823
1773
2699
1627
1962
1601
1994

LOG Ave Rt of Incorrect Colour Word (INCONGRUENT) Responses b +1
3,203576775
3,049218023
3,2509077
3,247482261
3,18920949
0
3,121231455
0
3,273001272
3,06595298
3,036628895
0
0
3,020775488
3,396024897
3,164055292
0
3,329397879
0
3,241048151
3,278982117
0
0
3,316389751
3,248953615
3,182129214
0
3,200850498
3,317854489
3,121559844
3,18610838
3,192846115
3,240798771
3,34321159
0
3,336259552
3,35545152
0
3,260786669
3,248708736
3,431202885
3,211387553
3,292699003
3,204391332
3,299725154

Ave Rt of Incorrect Grey Word (NEUTRAL) Responses a
699
1182
0
2802
1849
1826
0
2506
0
1073
0
0
1487
0
1878
1619
0
1633
0
1419
1678
0
0
1093
0
1284
1178
673
2676
1261
0
0
1766
830
0
0
0
1292
1183
0
0
2348
0
1454
0

Ave Rt of Incorrect Grey Word (NEUTRAL) Responses a +1
700
1183
1
2803
1850
1827
1
2507
1
1074
1
1
1488
1
1879
1620
1
1634
1
1420
1679
1
1
1094
1
1285
1179
674
2677
1262
1
1
1767
831
1
1
1
1293
1184
1
1
2349
1
1455
1

LOG Ave Rt of Incorrect Grey Word (NEUTRAL) Responses a +1
2,84509804
3,072984745
0
3,447623098
3,267171728
3,261738547
0
3,399154334
0
3,031004281
0
0
3,172602931
0
3,27392678
3,209515015
0
3,213252052
0
3,152288344
3,225050696
0
0
3,039017322
0
3,108903128
3,071513805
2,828659897
3,427648371
3,101059355
0
0
3,24723655
2,919601024
0
0
0
3,111598525
3,073351702
0
0
3,370883017
0
3,162862993
0

Ave Rt of Incorrect Grey Word (NEUTRAL) Responses b
2094
1636
1951
2551
1270
1009
0
0
2235
0
0
0
0
0
2047
0
2026
1881
0
1085
0
0
0
0
2534
0
0
1433
0
930
2529
0
1442
2018
0
1353
1992
0
0
0
0
1949
0
1570
862

Ave Rt of Incorrect Grey Word (NEUTRAL) Responses b +1
2095
1637
1952
2552
1271
1010
1
1
2236
1
1
1
1
1
2048
1
2027
1882
1
1086
1
1
1
1
2535
1
1
1434
1
931
2530
1
1443
2019
1
1354
1993
1
1
1
1
1950
1
1571
863

LOG Ave Rt of Incorrect Grey Word (NEUTRAL) Responses b +1	0Back.Score a	LOG 0 Back.Scorea
3,321184027	98	1,991226076
3,214048679	98	1,991226076
3,290479813	97	1,986771734
3,40688067	99	1,995635195
3,104145551	96	1,982271233
3,004321374	98	1,991226076
0	98	1,991226076
0	97	1,986771734
3,349471799	94	1,973127854
0	98	1,991226076
0	99	1,995635195
0	96	1,982271233
0	99	1,995635195
0	97	1,986771734
3,311329952	95	1,977723605
0	100	2
3,306853749	95	1,977723605
3,274619619	94	1,973127854
0	98	1,991226076
3,035829825	98	1,991226076
0	98	1,991226076
0	98	1,991226076
0	98	1,991226076
0	92	1,963787827
3,403977964	92	1,963787827
0	99	1,995635195
0	97	1,986771734
3,156549151	97	1,986771734
0	94	1,973127854
2,968949681	100	2
3,403120521	97	1,986771734
0	99	1,995635195
3,159266331	94	1,973127854
3,305136319	88	1,944482672
0	100	2
3,131618664	96	1,982271233
3,299507299	96	1,982271233
0	97	1,986771734
0	99	1,995635195
0	100	2
0	98	1,991226076
3,290034611	91	1,959041392
0	98	1,991226076
3,196176185	99	1,995635195
2,936010796	98	1,991226076

0Back.Score b	LOG 0 Back.Scoreb	0Back.Found.Targ a	0Back.Found.Targ b	0Back.Missed.Targ a
98	1,991226076	134	134	1
100	2	133	135	2
99	1,995635195	133	135	2
100	2	135	135	0
98	1,991226076	131	133	4
99	1,995635195	134	135	1
99	1,995635195	134	134	1
99	1,995635195	131	134	4
98	1,991226076	133	134	2
100	2	135	135	0
100	2	135	135	0
97	1,986771734	133	132	3
98	1,991226076	135	135	0
99	1,995635195	134	134	1
99	1,995635195	131	134	4
100	2	136	135	0
99	1,995635195	130	135	5
95	1,977723605	128	130	7
99	1,995635195	133	134	2
100	2	134	135	1
98	1,991226076	134	134	1
100	2	133	135	2
98	1,991226076	133	134	2
98	1,991226076	126	134	9
96	1,982271233	127	131	8
100	2	135	135	0
98	1,991226076	132	134	3
97	1,986771734	132	132	3
98	1,991226076	130	134	5
100	2	135	135	0
92	1,963787827	134	126	1
99	1,995635195	134	135	1
97	1,986771734	129	132	6
96	1,982271233	121	132	14
98	1,991226076	135	134	0
96	1,982271233	131	131	4
99	1,995635195	132	134	4
99	1,995635195	133	135	2
99	1,995635195	135	135	0
99	1,995635195	135	134	0
95	1,977723605	134	132	1
93	1,968482949	126	130	9
95	1,977723605	133	131	2
100	2	135	135	0
98	1,991226076	134	134	1

0Back.Missed.Targ b	0Back.Wrong.Targ a	0Back.Wrong.Targ b	0Back.Total.Targ a
1	1	1	135
0	0	0	135
0	4	1	135
0	1	0	135
2	1	0	135
0	1	2	135
1	2	0	135
1	0	0	135
1	12	2	135
0	5	0	135
0	1	0	135
3	3	1	136
0	2	3	135
1	6	0	135
1	3	0	135
0	0	0	136
0	1	1	135
6	2	0	135
1	0	0	135
0	1	0	135
1	1	2	135
0	0	0	135
1	0	1	135
1	1	1	135
4	3	2	135
0	1	0	135
1	1	1	135
3	0	1	135
1	4	1	135
0	0	0	135
9	4	3	135
0	0	1	135
3	2	0	135
3	4	4	135
1	0	1	135
5	1	0	135
1	2	0	136
0	2	1	135
0	2	2	135
1	0	0	135
5	1	1	135
5	4	7	135
4	1	3	135
0	1	0	135
1	3	3	135

0Back.Total.Targ b	1Back.Score a	LOG 1 Back.Score a	1Back.Score b	LOG 1 Back.Score b
135	95	1,977723605	98	1,991226076
135	97	1,986771734	100	2
135	92	1,963787827	90	1,954242509
135	86	1,934498451	98	1,991226076
135	90	1,954242509	88	1,944482672
135	100	2	98	1,991226076
135	99	1,995635195	97	1,986771734
135	98	1,991226076	98	1,991226076
135	95	1,977723605	98	1,991226076
135	97	1,986771734	94	1,973127854
135	98	1,991226076	95	1,977723605
135	97	1,986771734	95	1,977723605
135	98	1,991226076	97	1,986771734
135	95	1,977723605	95	1,977723605
135	94	1,973127854	98	1,991226076
135	96	1,982271233	99	1,995635195
135	89	1,949390007	97	1,986771734
135	74	1,86923172	93	1,968482949
135	98	1,991226076	100	2
135	93	1,968482949	98	1,991226076
135	92	1,963787827	95	1,977723605
135	97	1,986771734	97	1,986771734
135	98	1,991226076	97	1,986771734
135	100	2	98	1,991226076
135	78	1,892094603	75	1,875061263
135	99	1,995635195	98	1,991226076
135	100	2	100	2
135	73	1,86332286	85	1,929418926
135	93	1,968482949	79	1,897627091
135	100	2	97	1,986771734
135	93	1,968482949	90	1,954242509
135	100	2	100	2
135	93	1,968482949	93	1,968482949
135	82	1,913813852	100	2
135	92	1,963787827	100	2
136	82	1,913813852	84	1,924279286
135	90	1,954242509	92	1,963787827
135	95	1,977723605	98	1,991226076
135	97	1,986771734	96	1,982271233
135	96	1,982271233	100	2
137	93	1,968482949	96	1,982271233
135	90	1,954242509	98	1,991226076
135	100	2	100	2
135	99	1,995635195	100	2
135	99	1,995635195	96	1,982271233

1Back.Found.Targ a	1Back.Found.Targ b	1Back.Missed.Targ a	1Back.Missed.Targ b
60	66	3	0
69	61	0	0
63	63	4	4
57	61	8	1
56	59	5	4
62	61	0	1
60	66	0	1
60	60	1	1
58	65	2	1
62	58	1	1
62	57	0	2
63	65	1	1
64	62	1	1
59	58	2	2
58	66	2	1
62	67	2	0
58	64	4	1
48	59	15	2
64	64	1	0
62	64	2	0
56	59	4	2
67	62	1	0
63	68	0	0
62	57	0	1
51	49	10	16
64	60	0	1
57	63	0	0
48	53	15	8
61	48	1	10
67	65	0	0
63	58	3	4
65	61	0	0
57	60	2	2
54	71	10	0
57	57	3	0
53	52	10	9
58	52	5	4
60	62	2	1
60	63	1	1
64	65	1	0
58	61	3	2
58	60	5	1
66	64	0	0
61	65	0	0
60	62	0	2

1Back.Wrong.Targ a	1Back.Wrong.Targ b	1Back.Total.Targ a	1Back.Total.Targ b	2Back.Score a
0	2	63	66	29
3	0	69	61	63
2	5	67	67	62
2	0	65	62	79
1	6	61	63	74
0	0	62	62	58
1	2	60	67	51
0	0	61	61	90
2	0	60	66	68
1	4	63	59	61
2	1	62	59	81
1	4	64	66	50
0	1	65	63	64
1	2	61	60	58
3	0	60	67	66
1	1	64	67	92
5	1	62	65	75
2	4	63	61	0
0	0	65	64	20
4	2	64	64	76
1	2	60	61	49
2	3	68	62	67
2	4	63	68	84
0	0	62	58	58
6	0	61	65	0
1	0	64	61	92
0	0	57	63	70
3	2	63	61	73
6	4	62	58	37
0	3	67	65	79
2	4	66	62	0
0	0	65	61	87
4	4	59	62	37
2	0	64	71	2
3	0	60	57	83
2	1	63	61	46
2	0	63	56	0
1	0	62	63	70
1	3	61	64	78
3	0	65	65	66
2	1	61	63	61
2	0	63	61	23
0	0	66	64	11
1	0	61	65	68
1	1	60	64	60

2Back.Score a +1	LOG 2 Back.Score a	2Back.Score b	2Back.Score b +1	LOG 2 Back.Score b
30	1,477121255	50	51	1,707570176
64	1,806179974	64	65	1,812913357
63	1,799340549	78	79	1,897627091
80	1,903089987	72	73	1,86332286
75	1,875061263	64	65	1,812913357
59	1,770852012	62	63	1,799340549
52	1,716003344	76	77	1,886490725
91	1,959041392	96	97	1,986771734
69	1,838849091	74	75	1,875061263
62	1,792391689	91	92	1,963787827
82	1,913813852	85	86	1,934498451
51	1,707570176	90	91	1,959041392
65	1,812913357	88	89	1,949390007
59	1,770852012	74	75	1,875061263
67	1,826074803	82	83	1,919078092
93	1,968482949	79	80	1,903089987
76	1,880813592	83	84	1,924279286
1	0	22	23	1,361727836
21	1,322219295	68	69	1,838849091
77	1,886490725	92	93	1,968482949
50	1,698970004	57	58	1,763427994
68	1,832508913	88	89	1,949390007
85	1,929418926	98	99	1,995635195
59	1,770852012	64	65	1,812913357
1	0	60	61	1,785329835
93	1,968482949	87	88	1,944482672
71	1,851258349	94	95	1,977723605
74	1,86923172	89	90	1,954242509
38	1,579783597	83	84	1,924279286
80	1,903089987	85	86	1,934498451
1	0	38	39	1,591064607
88	1,944482672	85	86	1,934498451
38	1,579783597	91	92	1,963787827
3	0,477121255	71	72	1,857332496
84	1,924279286	94	95	1,977723605
47	1,672097858	38	39	1,591064607
1	0	95	96	1,982271233
71	1,851258349	45	46	1,662757832
79	1,897627091	90	91	1,959041392
67	1,826074803	71	72	1,857332496
62	1,792391689	78	79	1,897627091
24	1,380211242	48	49	1,69019608
12	1,079181246	51	52	1,716003344
69	1,838849091	62	63	1,799340549
61	1,785329835	68	69	1,838849091

2Back.Found.Targ a	2Back.Found.Targ b	2Back.Missed.Targ a	2Back.Missed.Targ b
35	41	19	16
51	47	9	10
37	45	14	8
49	41	9	11
45	50	8	7
44	44	13	12
35	46	18	8
51	53	4	2
45	44	13	11
44	55	7	3
47	50	9	6
38	51	14	4
44	57	12	2
38	40	17	10
44	44	15	8
52	47	4	8
43	48	11	6
27	31	28	27
43	53	10	2
48	54	9	3
36	33	20	22
38	49	18	5
56	54	3	0
42	39	15	13
18	34	37	19
52	49	2	5
41	53	15	3
46	50	9	5
26	50	27	5
49	53	4	6
4	26	48	32
57	46	1	5
25	54	29	4
27	54	27	3
49	57	7	2
38	43	18	13
48	56	10	2
41	36	12	20
51	54	8	4
46	45	10	11
43	52	13	6
42	44	13	9
23	31	33	23
49	42	6	13
40	44	16	11

2Back.Wrong.Targ a	2Back.Wrong.Targ b	2Back.Total.Targ a	2Back.Total.Targ b	Clock.Draw a
38	25	54	57	2
26	20	60	57	2
10	7	51	53	1
6	7	58	52	2
11	27	53	57	2
21	18	57	56	3
15	9	53	54	2
3	0	55	55	4
11	6	58	55	2
25	4	51	58	2
3	4	56	56	1
23	3	52	55	2
16	10	56	59	1
12	6	55	50	1
9	2	59	52	2
0	7	56	55	2
5	6	54	54	4
82	36	55	58	6
64	31	53	55	1
9	2	57	57	2
17	3	56	55	2
0	2	56	54	2
12	2	59	54	1
17	11	57	52	3
109	4	55	53	3
4	3	54	54	3
3	0	56	56	4
11	2	55	55	1
12	8	53	55	3
14	5	53	59	1
27	7	52	58	3
13	5	5	51	1
9	2	54	58	6
51	27	54	57	4
4	2	56	59	2
24	43	56	56	2
181	1	58	58	1
7	21	53	56	2
9	3	59	58	2
18	10	56	56	2
17	13	56	58	2
58	37	55	53	2
33	6	56	54	2
23	15	55	55	2
12	13	56	55	2

Clock.Draw b	CLK Draw Difference (a-b)	MMSEa	MMSEb
2	0	19	23
2	0	27	28
2	-1	22	29
1	1	27	28
2	0	24	25
1	2	25	28
2	0	25	28
1	3	26	22
1	1	26	29
2	0	29	29
1	0	28	30
1	1	21	25
1	0	23	26
2	-1	23	26
4	-2	21	29
2	0	25	29
2	2	25	28
4	2	23	25
1	0	26	28
1	1	25	30
1	1	22	28
2	0	25	29
2	-1	30	29
2	1	28	23
5	-2	24	24
2	1	24	26
2	2	25	28
1	0	30	28
2	1	25	22
1	0	28	28
2	1	23	22
1	0	25	28
5	1	20	20
3	1	23	22
1	1	26	27
2	0	19	21
4	-3	26	25
2	0	26	26
2	0	26	29
3	-1	24	28
2	0	24	26
1	1	25	23
2	0	25	27
2	0	28	24
2	0	25	27

Are you currently employed (Yes=1 ; No=0)	Job
0	0
0	0
0	0
0	0
0	0
0	0
0	0
1	Nurse
0	0
0	0
0	0
0	0
0	0
0	0
0	0
0	0
0	0
0	0
0	0
0	0
0	0
0	0
0	0
0	0
0	0
0	0
0	0
1	Morning Carer
0	0
0	0
0	0
0	0
0	0
0	0
0	0
0	0
0	0
0	0
0	0
0	0
0	0
0	0
1	Merchandiser
0	0
0	0

Current Marital Status (Married=1; Single=2; Divorced=3; Widowed=4)	School	Tertiary	Total
3	9	0	9
4	7	0	7
4	7	0	7
4	10	0	10
4	8	0	8
3	12	5	17
4	12	2	14
1	10	3	13
3	12	3	15
4	12	0	12
4	12	2	14
3	7	0	7
1	10	0	10
1	8	0	8
4	12	1	13
4	11	0	11
2	7	0	7
4	7	0	7
1	8	0	8
2	10	4	14
4	5	0	5
4	5	0	5
4	11	0	11
2	9	0	9
3	9	0	9
4	8	0	8
3	9	3	12
4	12	4	16
1	11	0	11
1	10	0	10
1	8	0	8
1	10	3	13
1	9	0	9
3	7	0	7
1	10	4	14
1	7	0	7
4	7,5	0	7,5
4	10	0	10
4	10	0	10
3	9	0	9
4	8	0	8
3	12	2	14
4	10	0	10
3	8	0	8
4	6	0	6

Number of Years at Current Address
8
14
1
2,58
0,16
0,33
4
7
3
4
2,42
0,375
0,083
0,083
3,5
5,42
5,25
10,92
8,75
7,58
8,58
5,5
6,42
2
5,17
5,33
4
11,58
14,25
5
8
7,25
10
2
2,7
10
8
2
10
2,5
15
3,5
2,83
22
1,5

Highest Level of Education Obtained (Primary=1; Secondary=2; Tertiary=3)
1
1
1
1
1
3
3
3
3
2
2
1
1
1
3
1
1
1
1
3
1
1
1
1
1
1
3
3
1
1
1
3
1
1
3
1
1
1
1
1
1
3
1
1
1

Please Rate Your Quality of Health (Very Poor=1; Poor=2; Average=3; Good=4; Excellent=5)
4
4
3
3
3
4
4
3
4
3
3
3
4
4
3
4
4
5
3
4
4
4
4
3
3
3
3
4
3
4
3
4
4
4
4
3
2
4
4
4
5
3
5
4
4

Health Better, Same or Worse Than Others (Better=1; Same=2; Worse=3)	Hypertension
1	1
1	1
2	1
1	0
1	1
1	1
2	1
1	0
1	0
1	1
1	0
1	1
1	1
1	1
2	1
1	1
1	1
1	1
2	1
1	1
1	1
1	1
1	0
1	1
2	1
1	0
1	1
1	1
1	0
1	1
2	0
1	1
1	0
1	0
1	1
3	1
2	1
1	1
1	0
1	1
1	0
1	1
1	1
1	1
2	1

High Cholesterol	Diabetes	Asthma	PVD	None	Are You On Medication (Yes=1; No=0)
0	1	0	0	0	1
1	1	0	0	0	1
1	0	1	0	0	1
0	1	0	0	0	1
1	1	0	0	0	1
1	1	1	1	0	1
1	1	0	0	0	1
0	0	0	0	1	0
0	0	0	0	1	0
1	0	0	0	0	1
1	0	0	0	0	1
0	1	0	0	0	1
0	0	0	0	0	1
0	0	0	0	0	1
1	1	0	0	0	1
0	0	0	0	0	1
1	0	0	0	0	1
1	0	0	0	0	1
1	1	0	0	0	1
0	0	0	0	0	1
1	1	0	0	0	1
0	0	0	0	0	1
1	0	1	0	0	1
1	0	0	0	0	1
0	0	0	0	0	1
0	0	0	0	0	1
1	0	0	0	0	1
0	0	0	0	0	1
0	0	0	0	1	1
0	0	0	0	0	1
0	1	0	0	0	1
1	1	0	0	0	1
1	0	0	0	0	1
1	0	0	0	0	1
1	1	0	0	0	1
1	0	0	0	0	1
1	0	0	0	0	1
0	0	0	0	0	1
1	0	0	0	0	1
0	0	0	0	0	1
1	1	0	0	0	1
1	0	0	0	0	1
0	0	0	0	0	1
0	0	0	0	0	1
0	0	0	0	0	0

TOTAL HOUSE WORK E.E	TOTAL YARD WORK E.E	TOTAL CARE TAKING E.E	TOTAL EXERCISE E.E
717,5	0	660	0
1034,5	0	0	240
1977,5	176,125	0	630
2585	0	0	0
742,5	0	0	0
3775	75	0	630
457,5	0,75	0	0
965	0	27,5	0
752,5	1,125	0	1620
2022,5	0	480	0
1205	100	0	135
1310	150	0	0
3342,5	2,25	2215	240
3687,5	1140	0	0
1348,5	0	0	1800
1076	0	0	0
2174	0	0	0
2704,5	0	27,5	0
1397,5	0	0	630
2364	0	0	750
4321,5	0	0	210
2985	0	0	0
1488	0	0	0
920	0	0	0
1361,5	0	0	0
2538,5	0	0	450
1280	0	0	360
1212,5	270	0	0
1732,5	957,5	0	270
0	0	0	720
205	300	0	0
1167,5	270	0	0
1129	10	0	840
1777,5	1080	0	0
9365	4080	0	240
3000	300	0	0
1094	0	720	0
2565,5	0	295	120
572,5	225	0	240
3109,5	615	0	375
3022,5	45	0	90
2550	350	0	0
2755	870	0	0
1225	180	0	0
2755	495	0	240

TOTAL RECREATION E.E	TOTAL E.E	TOTAL P.A MINS
1470	2847,5	785
35	1309,5	441
312,5	3096,125	915
505	3090	1010
245	987,5	355
1027,5	5507,5	1510
210	668,25	240
0	992,5	335
0	2373,625	630
960	3462,5	1270
210	1650	525
137,5	1597,5	441
825	6624,75	1915
420	5247,5	1235
735	3883,5	935
645	1721	536
210	2384	834
630	3362	1088
950	2977,5	805
1920	5034	1834
6300	10831,5	5239
3670	6655	3050
0	1488	498
370	1290	540
490	1851,5	555
315	3303,5	1036
82,5	1722,5	465
2100	3582,5	1480
900	3860	1200
35	755	130
1935	2440	830
280	1717,5	510
720	2699	706
3780	6637,5	2385
595	14280	4265
1142,5	4442,5	1415
1640	3454	1204
442,5	3423	1178
97,5	1135	345
162,5	4262	1307
470	3627,5	1220
210	3110	1030
1085	4710	1275
990	2395	740
70	3560	1220
