# Supplementary material for: Efficacy of interactive video gaming in older adults with memory complaints: A cluster-randomized exercise intervention
Source: PLoS One. 2021 May 25;16(5):e0252016. doi: 10.1371/journal.pone.0252016 (PMC8148311; doi:10.1371/journal.pone.0252016)
Supplement: S1 Questionnaire — (PDF) [file pone.0252016.s003.pdf]

THE DEMOGRAPHIC, HEALTH and WELLNESS QUESTIONNAIRE

PERSONAL DETAILS	
Name:	Surname:
Age:	Date of Birth:
Code:	Date:

Section One: Socio-demographic and health information

1) Sex:	1. Male		2. Female	

2) Are you currently employed?			
1. Yes		2. No	
If yes, what job do you do?			

3) What is your current marital status?	
1. Married	
2. Single	
3. Divorced	
4. Widowed	

4) How many years of education did you complete?	

5) How long are you living at this current address / retirement village?

6) What is your highest level of education obtained?	
1. Primary School Education	
2. Secondary School Education	
3. Tertiary Education	

7) How would you rate your current health status?	
1. Very poor	
2. Poor	
3. Average	
4. Good	
5. Excellent	

8) Do you think your health is Better, the Same or Worse than/as others the same age as you?	
1. Better	
2. Same	
3. Worse	

9) Has your doctor previously diagnosed you for any of the following medical conditions:		Are you on medication?		
		Yes		No
1. Hypertension		Name:		
		Dosage:		
2. High cholesterol		Name:		
		Dosage:		
3. Diabetes mellitus		Name:		
		Dosage:		
4. Asthma		Name:		
		Dosage:		
5. Peripheral vascular disease		Name:		
		Dosage:		
6. None				

Section Two: Weekly Physical Activity Habits

**Record of appointment and time**	**Date:**	**Time:**

This questionnaire was developed for the use on older adults. We are using this questionnaire to calculate the amount for physical activity you participate in during a week and what is your total energy expenditure from the activities you participate in. The questions are based on 5 categories such as: ***work, yard work, care taking, exercise and recreation.***

**ACTIVITY**	**TIME**			**INTENSITY CODE**
	**hours**	**minutes**	**Days**	
**WORK**				
Shopping (eg, clothes, groceries)				3.5
Stair Climbing while carrying a load				8.5
Laundry: unloading/loading machine, hanging, folding only,				3.0
washing clothes by hand				4.0
Light housework: tidying, dusting, sweeping, collecting rubbish in the home, polishing, ironing.				3.0
Heavy housework: vacuuming, mopping, scrubbing floors and				4.5

walls, moving furniture, boxes or rubbish bins				
Food preparation: chopping, stirring, moving about to get food items and pans.				2.5
Food services: setting table, carrying food, serving food				2.5
Dish washing: clearing the table, washing/drying dishes, putting dishes away.				2.5
Light home repairs. Small appliance repairs light home maintenance / repair.				3.0
Heavy home repairs: painting, carpentry, washing/polishing car.				5.5
Other:				

**ACTIVITY**	**TIME**			**INTENSITY CODE**
	**hours**	**minutes**	**days**	
**YARD WORK**				
Gardening, pruning, planting, weeding, digging, hoeing				4.5
Lawn mowing (walking only)				4.5
Clearing walks / driveways : sweeping, shovelling, raking				5.0
Other:				

ACTIVITY	TIME			INTENSITY CODE
	hours	minutes	days	
**CARE TAKING**				
Older or disabled person (lifting, pushing wheelchair)				5.5
Child care (lifting, carrying, pushing pram)				4.0

ACTIVITY	TIME			INTENSITY CODE
	hours	minutes	days	
**EXERCISE**				
Brisk walking				6.0
Pool exercises, stretching, yoga				3.0
Vigorous calisthenics, aerobics				6.0
Cycling				6.0
Swimming (laps only)				6.0
Other				

ACTIVITY	TIME			INTENSITY CODE
	hours	minutes	days	
**RECREATION**				
Leisurely / slow walking				3.5
Needlework: knitting, sewing, needlepoint etc.				1.5
Dancing: line, ballroom, tap, square etc.				5.5
Bowling				3.0
Golf				5.0
Racquet sports: tennis, squash				7.0
Billiards				2.5
Other				
